# Identifying an Inversin as a Novel Prognostic Marker in Patients with Clear-Cell Renal Cell Carcinoma

**DOI:** 10.3390/ijms252212120

**Published:** 2024-11-12

**Authors:** Ivanka Urlić, Violeta Šoljić, Martina Vukoja, Inga Marijanović, Marija Kraljević, Marjan Urlić, Sara Marić, Katarina Vukojević, Natalija Filipović

**Affiliations:** 1 Department of Oncology and Radiotherapy, University Hospital of Split, 21000 Split, Croatia; 2 Laboratory of Morphology, Department of Histology and Embryology, School of Medicine, University of Mostar, 88000 Mostar, Bosnia and Herzegovina; vsoljic@gmail.com (V.Š.); martina.vukoja@mef.sum.ba (M.V.); kvukojev@gmail.com (K.V.); 3 Faculty of Health Studies, University of Mostar, 88000 Mostar, Bosnia and Herzegovina; 4 Clinic of Oncology, University Clinical Hospital Mostar, 88000 Mostar, Bosnia and Herzegovina; inga.marijanovic@mef.sum.ba (I.M.); marija.kraljevicc91@gmail.com (M.K.); 5 Department of Cardiac Surgery, University Hospital Centre Zagreb, 10000 Zagreb, Croatia; urlicmarjan@gmail.com; 6 Odjel za Patološku Anatomiju i Citologiju, Kantonalna Bolnica Dr. Safet Mujić, 88000 Mostar, Bosnia and Herzegovina; sdozlic@gmail.com; 7 Department of Anatomy, Histology and Embryology, University of Split School of Medicine, 21000 Split, Croatia

**Keywords:** clear-cell renal cell carcinoma, inversin, interactome, prognosis, target

## Abstract

Precision medicine is a developing trend in oncology, and it includes the prognosis and treatment of advanced-stage ccRCC. New predictive factors and therapeutic targets for this disease are steadily needed. The aim of this study was to explore the tumor expression of inversin as a potential prognostic factor and/or therapeutic target in ccRCC. We compared the expression of inversin between primary ccRCC and normal renal tissues by using immunohistochemistry and rtPCR in our cohort, and we also analyzed publicly available data from the TCGA-KIRC cohort. We found that the expression of inversin was significantly lower in primary tumor tissue, in comparison to solid normal tissue. Data from the KIRC study confirmed that a lower *INVS* expression level in ccRCC was significantly related with the overall and disease-specific survival, as well as with a shorter progression-free interval (*p* < 0.05). Four out of ten inversin interactome partners were significantly related with the overall and disease-specific survival in ccRCC. A lower expression of *ANKS6* was a negative survival predictor, while a higher expression of *NPHP3*, *DVL1*, or *DVL3* was related with a lower survival. The expression of *INVS* and its interactome partners in ccRCC was correlated with the differentiation of the tumor and metastasis. The expression of *INVS* and its partners was also correlated with tumor leukocyte infiltration and the expression of immune checkpoint genes. The results of this study point to inversin and a distinguished group of its interactome partners as potential prognostic factors in ccRCC, with their predominant involvement in the modulation of the inflammatory infiltration of the tumor microenvironment and a strong relationship with the metastatic potential of the tumor.

## 1. Introduction

Kidney cancer comprises approximately 2–3% of all new cancer cases. According to the International Agency for Research on Cancer and the World Health Organization, the worldwide incidence of kidney neoplasms was 432,463 cases in 2022 [1]. Approximately 85% of kidney tumors are renal cell carcinoma (RCC), and the most frequent histology type is clear-cell RCC (ccRCC), with an incidence of approximately 70%. Papillary, chromophobe, translocation, and Bellini duct tumors are other less common histology types [2]. The etiology of ccRCC is complex and involves some well-known risk factors, such as cigarette smoking, obesity, and hypertension, as well as several hereditary RCC syndromes that may predispose patients to a relatively rare hereditary type of RCC (around 3% of all RCC cases), with an autosomal-dominant von Hippel–Lindau (VHL) disease being the most common [2,3,4]. ccRCC is an extremely heterogeneous disease, with a different tumor biology. Many different factors have been found to be involved in ccRCC development and progression. Genomic alterations involving *SETD2*, *BAP1*, and *PBRM1* may be identified, or the deregulation of the mTOR pathway may occur due to mutations in the *MTOR*, *TSC1*, *PIKSCA*, or *PTEN* genes, thus affecting disease progression, the chance of aggressive phenotypes, and the patient’s prognosis [5]. The standard-of-care treatment for local and locoregional ccRCC is partial or radical nephrectomy [4]. According to prognostic risk factors (tumor histological subtype, size of the disease, nuclear grade, necrosis, and stage), different prognostic models have been developed to assess a patient’s individual risk for recurrence after surgery [6,7,8] and to identify candidates for the application of adjuvant immunotherapy [9]. Despite all of this, up to 50% of patients will eventually develop disease recurrence, mainly as distant metastases [8,9,10], resulting in ~175,000 deaths per year. Hence, there is a need for further investigations of additional prognostic indicators, which would be essential to guide individualized, risk-adapted surveillance protocols and patient counseling in localized and locoregional disease, with the aim of identifying those patients who will relapse.

It is known that the aberrant activation of the Wnt signaling pathway affects the behavior of cancer stem cells, playing a significant role in the initiation, maintenance, and/or development of many tumors [11,12]. The aberrant activation of the Wnt/planar cell polarity (PCP) signaling pathway in human cancer leads to more malignant phenotypes (invasion and metastasis) [12,13]. One of the main factors involved in the regulation of the Wnt signaling pathway is INVS (inversin; nephrocystin-2) [14,15]. INVS is a ciliary protein (encoded by *NPHP2*) that consists of 1065 amino acids. Also known as nephrocystin-2, it regulates developmental processes and has a role in tissue homeostasis and the coordination of cell migration [16]. INVS targets the degradation of cytoplasmatic dishevelled-1 (Dsh or Dvl1), increasing its ubiquitination. Through this mechanism, INVS maintains the inhibition of the canonical Wnt signaling pathway, sustaining low levels of a downstream component—the transcription factor B-catenin [14,15]. The role of INVS as a molecular switch between Wnt signaling cascades (canonical and noncanonical Wnt signaling) has an important impact on cell division, ciliary polarization, renal development, and cystogenesis [15]. INVS has a role in the noncanonical Wnt pathway as well. In an animal embryonic stage study, it was shown that the overexpression of INVS, as well as a knockdown of endogenous INVS, impaired convergent extension movements [15]. The absence of INVS resulted in the constitutive activation of the canonical Wnt pathway, which might be associated with abnormal primary cilia and cyst formation, leading to severe polycystic kidney diseases, multicystic dysplastic kidneys (MCDKs), focal segmental glomerulosclerosis (FSGS), or nephrotic syndrome of the Finnish type (CNF) [15,17].

Mutations in *INVS* cause nephronophthisis type II (an autosomal recessive cystic kidney disease characterized by extensive renal cysts, situs inversus, and renal failure) [18,19]. Concerning its role as one of the main factors involved in the regulation of the Wnt signaling pathway as well as its importance in renal development and the pathogenesis of different renal pathologies [14,15,16,17,18,19], the biological function of INVS in cancer is starting to be revealed. So far, it has been proven that INVS expression is correlated with the malignant phenotype of non-small cell lung cancer due to INVS enhancing the invasiveness of lung cancer cells, and it was identified as a poor prognostic factor [20]. However, there have been no studies on the expression and potential role of INVS in other cancer types, including kidney cancer. Clear-cell renal cell carcinoma is malignant with a high percentage of mortality due to the formation of distant metastases [21,22]. There is a growing need for novel prognostic indicators, which would be helpful in creating individualized surveillance protocols and in determining the prognosis of a relapse. Hence, the aim of the present study was to explore the potential role of INVS and its interactome partners in the pathogenesis and prognosis of ccRCC.

## 2. Results

### 2.1. INVS Is Expressed in Normal Renal Cells and Tumors, and Its Expression Is Decreased in ccRCC

**Figure 1:**
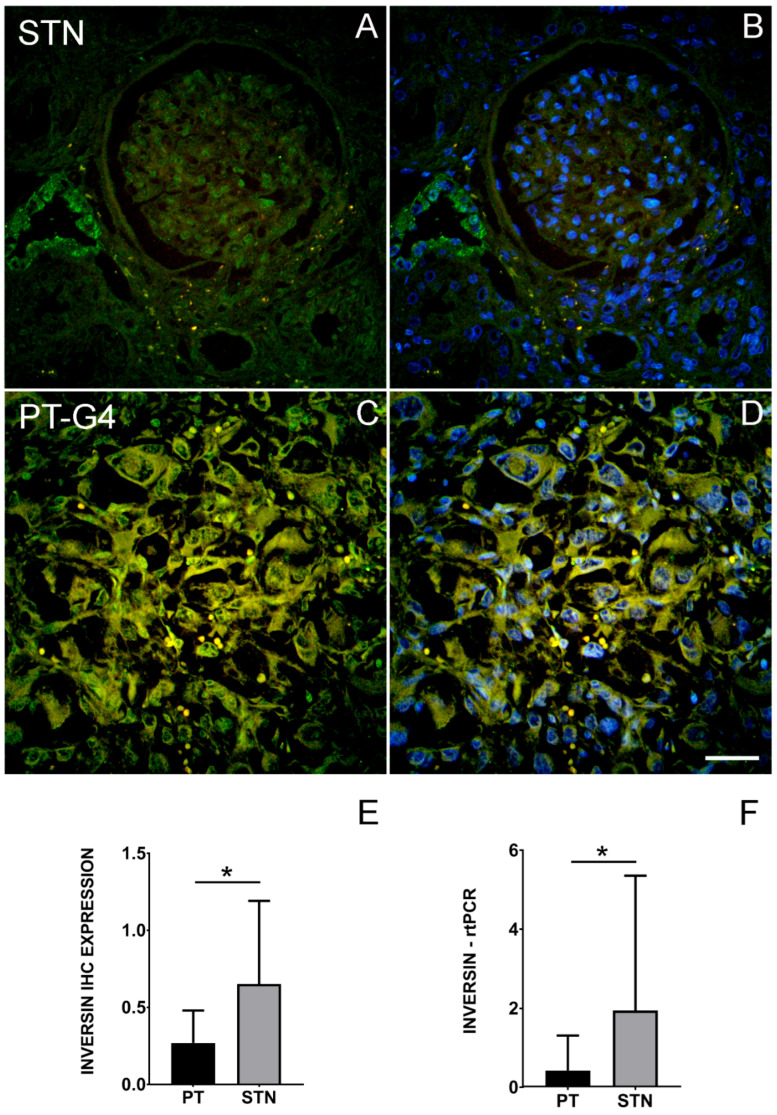
Representative immunofluorescence staining of INVS protein (green) in solid normal renal tissue (STN; (**A**,**B**)) and a primary tumor with a histological grade of 4 (PT-G4; (**C**,**D**)) (blue—DAPI; (**B**,**D**); magnification ×400). (**E**) Results of immunofluorescence quantification (INVS IHC expression in 34 ccRCC primary tumors (PTs) and 19 specimens of solid normal renal tissue (STN)). (**F**) Results of a real-time RT-PCR INVS analysis from 28 ccRCC PTs and 10 specimens of STN. * *p* < 0.05, indicating a difference between the experimental groups. Scale bar = 50 µm; refers to all.

We aimed to explore the potential role of INVS in ccRCC. By using the immunohistochemistry of primary ccRCC, we found in our cohort containing 34 patients with metastatic disease that the expression of INVS was significantly lower in primary tumors in comparison to solid normal tissue (Figure 1). In the normal tissue, the strongest INVS immunoreactivity was observed in the epithelial tubular cells, but it was also found to be present in glomeruli. The expression pattern was cytoplasmic and/or nuclear. In the tumor specimens, a weak, mostly nuclear expression was found. Data from the Human Protein Atlas confirmed a moderate to high expression of INVS mRNA and protein in different tissues, including the kidneys (Supplementary Figure S1). Data on a single-cell transcriptome analysis of the kidneys in the HPA from the GSE131685 dataset [23] confirmed the INVS mRNA expression in different clusters of epithelial cells, including the epithelium of the proximal and distal convoluted tubules as well as the collecting duct cells (Supplementary Figure S2). In addition, INVS mRNA was found to be present in different types of tumors, including ccRCC tumors, as well as in various cell lines of normal renal cells and renal tumor lines (Supplementary Figure S3).

### 2.2. INVS Expression in ccRCC Is Negatively Correlated with Patient Survival

**Figure 2:**
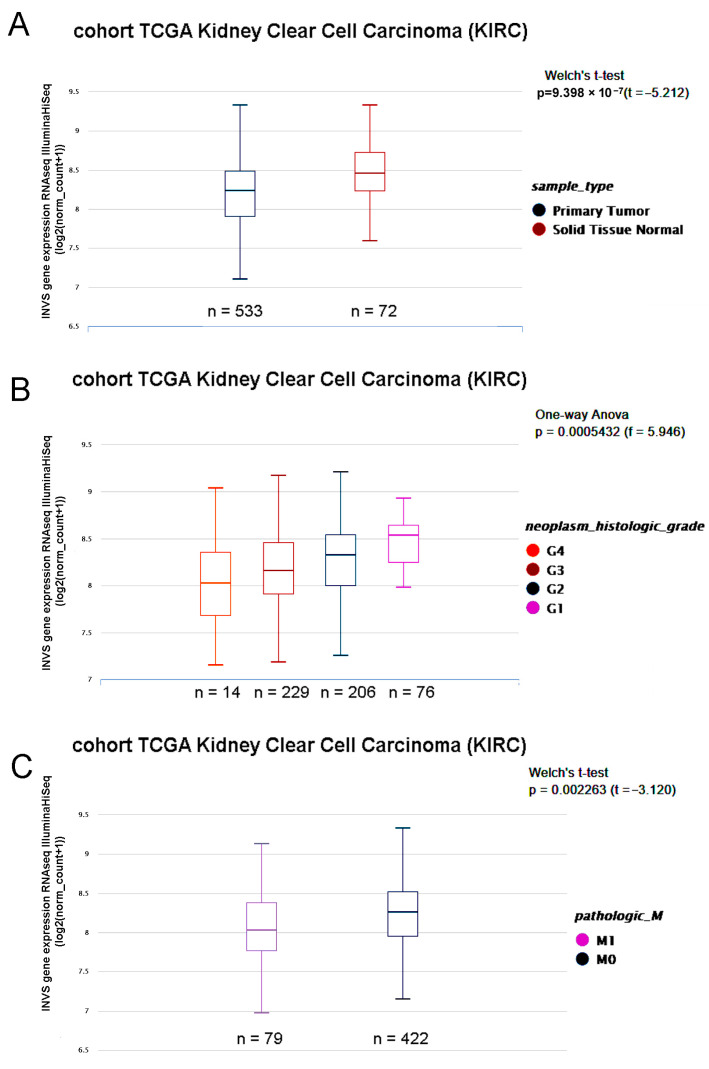
(**A**) The mRNA expression level of INVS in ccRCC primary tumors and adjacent solid normal tissues in TCGA-KIRC. (**B**) Comparison of the INVS mRNA expression in primary tumors from TCGA-KIRC between different neoplasm histological grades. (**C**) Comparison of the INVS mRNA expression in primary tumors from TCGA-KIRC between metastatic and non-metastatic tumors.

**Figure 3:**
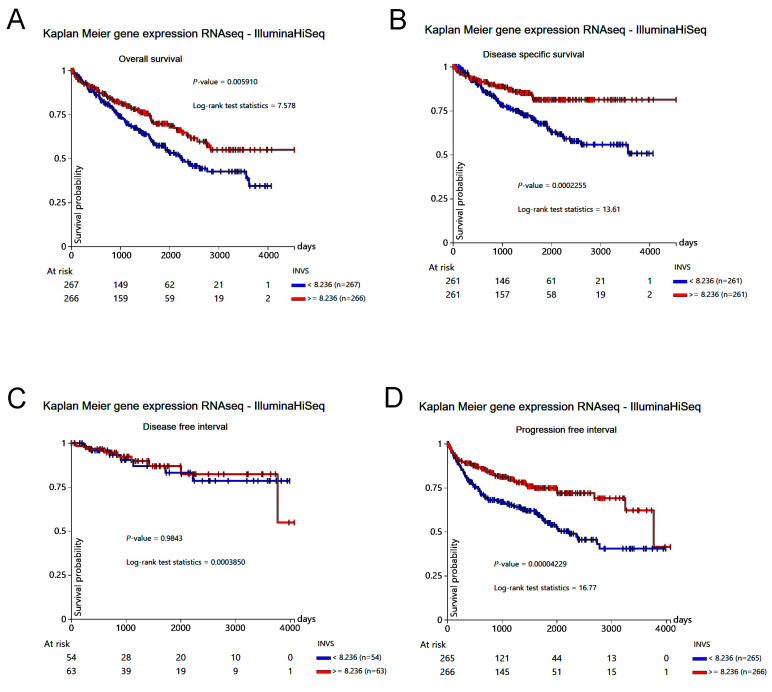
Kaplan–Meier curve analysis of overall survival (**A**), disease-specific survival (**B**), disease-free interval (**C**), and progression-free interval (**D**), grouped by INVS expression (using median as cutoff value) in TCGA-KIRC.

To support the data from our cohort, we explored the data from the KIRC study, which confirmed a significantly lower expression of INVS in primary tumors in comparison to solid normal tissue (Figure 2). A lower INVS expression was significantly related to the overall and disease-specific survival, as well as a shorter progression-free interval (Figure 3).

### 2.3. INVS Interactome Partners’ Expression in ccRCC Is Correlated with Patient Survival

**Figure 4:**
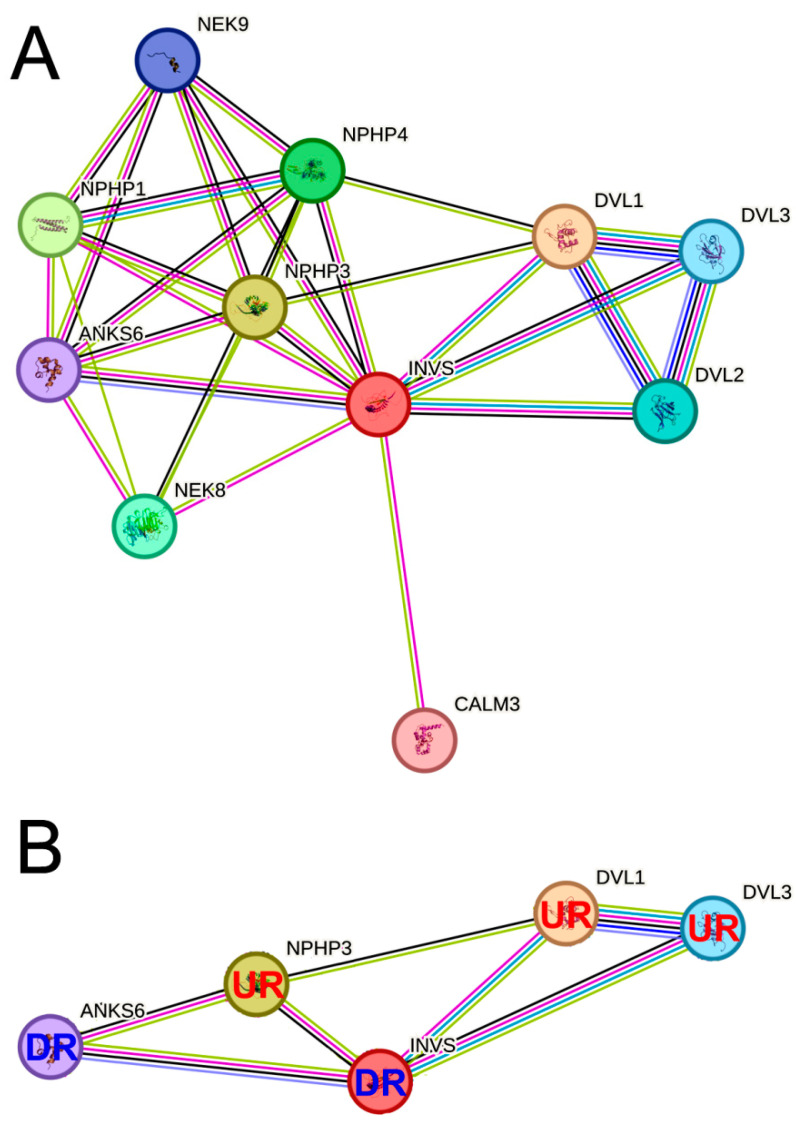
(**A**) INVS connectome network according to the STRING database. (**B**) INVS connectome network after removal of the connectome partners that did not significantly affect the patient survival probability in TCGA-KIRC. UR—a connectome partner whose upregulated expression is related to a lower probability of survival; DR—a connectome partner whose downregulated expression is related to a lower probability of survival.

**Figure 5:**
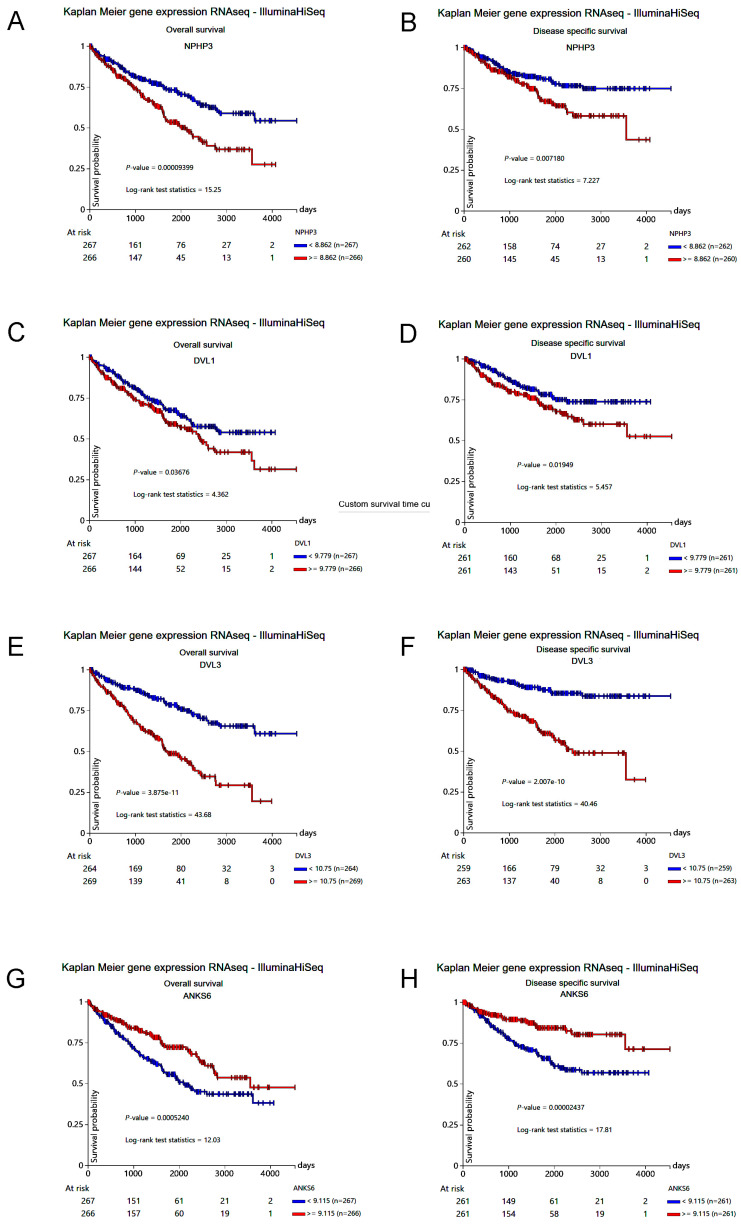
Kaplan–Meier curve analysis of overall survival (**A**,**C**,**E**,**G**) and disease-specific survival (**B**,**D**,**F**,**H**), grouped by the INVS interactome partner expression (using median as cutoff value) in TCGA-KIRC. Only significantly related partners are presented.

In order to reveal the potential pathogenetic role of INVS in ccRCC, we firstly explored the STRING database [24] to find INVS interactions with other proteins. Ten interactome partners were identified (Figure 4A): NPHP1, NPHP3, NPHP4, DVL1, DVL2, DVL3, ANKS6, NEK8, NEK9, and CALM3. The expression of four out of these ten partners in ccRCC, NPHP3, DVL1, DVL3, and ANKS6 was found to be significantly related to the overall and disease-specific survival (Figure 5). Similarly to the INVS, a lower expression of ANKS6 was a negative survival predictor, while a higher expression of NPHP3, DVL1, or DVL3 was related to a lower survival. Hence, we constructed a network of the INVS interactome relevant for the ccRCC outcome (Figure 4B) and included these four INVS interactome partners in the additional study. We found that the expression of NPHP3, DVL1, and DVL3 was significantly higher and the expression of ANKS6 was significantly lower in the primary tumor in comparison to the normal solid tissue (Supplementary Figure S4).

### 2.4. Expression of INVS and Its Interactome Partners in ccRCC Is Correlated with Differentiation of Tumor and Metastasis

By exploring the data from the TCGA-KIRC using the Xena USCC portal [25], we found that the INVS mRNA expression in primary tumors is inversely related to the neoplasm histological grade, being the highest in G1 and the lowest in G4 tumors. Moreover, the exploration of the data from the KIRC study revealed that the INVS expression was significantly lower in metastatic, in comparison to non-metastatic, ccRCC (Figure 2).

The expression of DVL3 was positively related and the expression of ANKS6 was negatively related to the tumor’s histological grade (Supplementary Figure S5). In addition, the expression of DVL3 was significantly higher, while the expression of ANKS6 was significantly lower in metastatic primary ccRCC, in comparison with the non-metastatic primary tumors (Supplementary Figure S6). Moreover, when we analyzed only a group of metastatic ccRCC tumors, the DVL3 was shown to be strongly predictive of patient survival (Supplementary Figure S7).

### 2.5. Potential Mechanism of INVS Correlation with ccRCC’s Clinical Outcome

**Figure 6:**
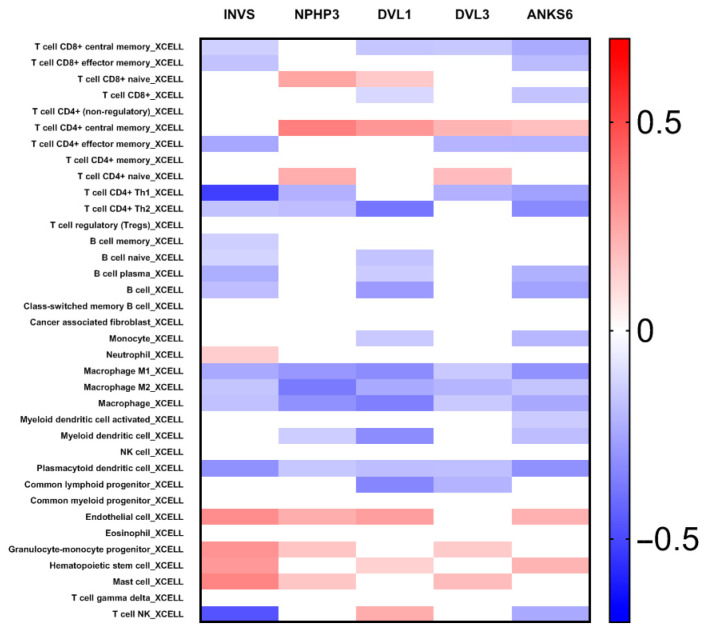
Correlation analysis of INVS and INVS interactome partner expression and abundance of tumor-infiltrating lymphocytes in TCGA-KIRC, according to the XCELL algorithm. Significant correlations are presented in color, while non-significant correlations (*p* > 0.05) are presented in white.

**Figure 7:**
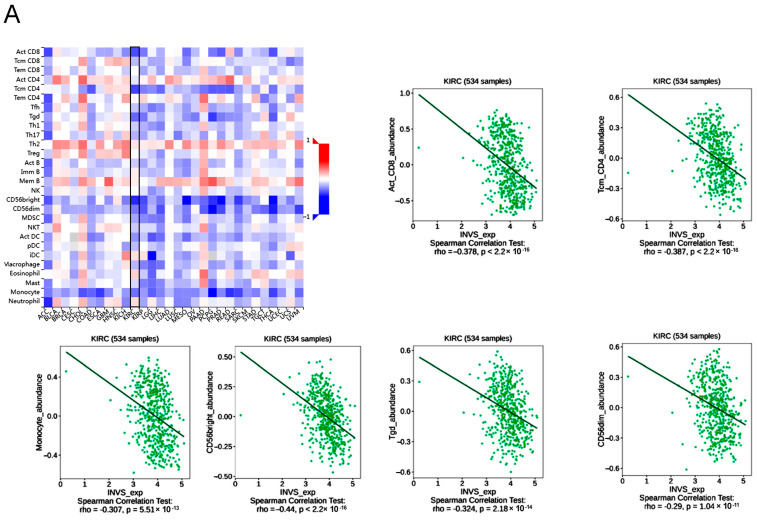

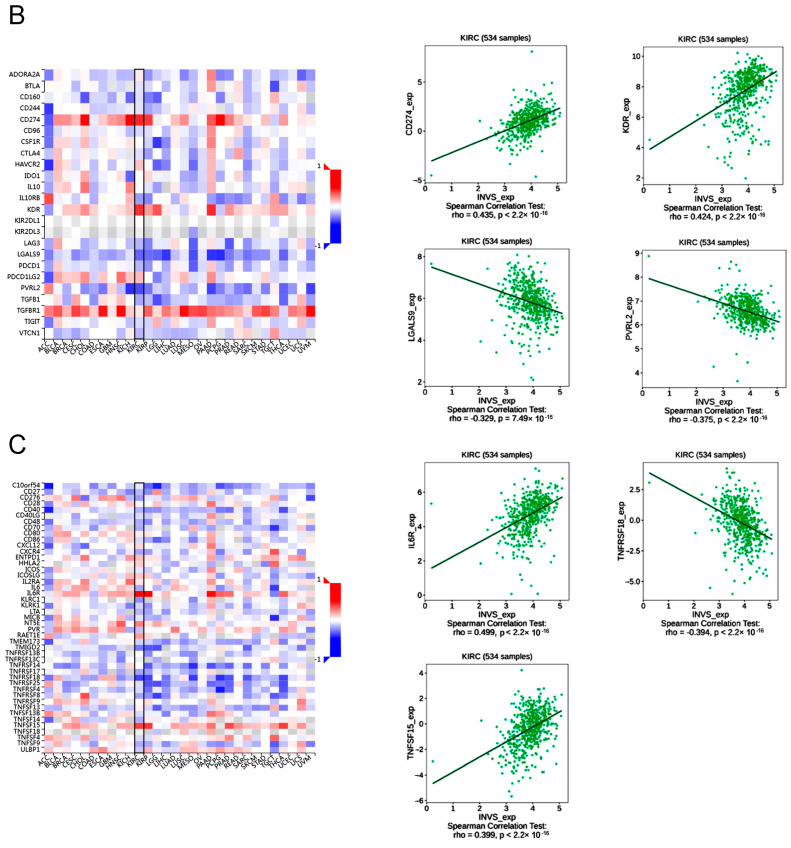
Correlation analysis of INVS expression and the abundance of tumor-infiltrating lymphocytes (**A**) in different cancers from TCGA (using the TISIDB database); the KIRC database is pointed out by using a black box. Significant correlations in the KIRC, with rho =/> 0.3 and rho </= −0.3, are presented as scatter plots. Correlation analysis of *INVS* expression and the immunoinhibitory (**B**) and immunostimulatory (**C**) gene expression in different cancers from TCGA (using the TISIDB database); the KIRC database is pointed out. Significant correlations in the KIRC, with rho =/> 0.3 and rho </= −0.3, are presented as scatter plots.

**Figure 8:**
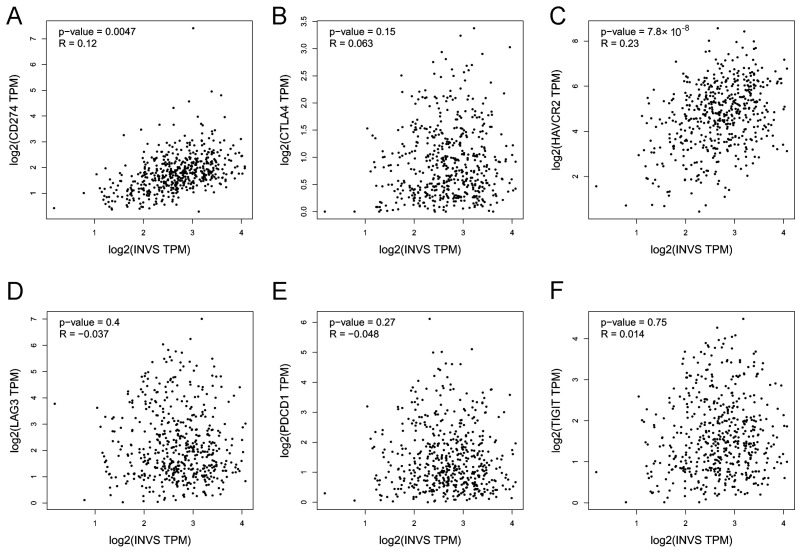
The correlation of the *INVS* expression with the immune checkpoint gene expression in KIRC using the GEPIA database—CD274 (**A**), CTLA4 (**B**), HAVCR2 (**C**), LAG3 (**D**), PDCD1 (**E**), and TIGIT (**F**). Positive significant correlations were found between the INVS expression and CD274 and HAVCR2.

In order to reveal the mechanism of INVS and its interactome effects on ccRCC pathogenesis and the clinical outcome, we explored the correlation of INVS and its partners with tumor leukocyte infiltration (Figure 6 and Figure 7; Supplementary Figures S8–S10) and the expression of immune checkpoint inhibitors, by using TISIDB [26] and GEPIA [27] portals (Figure 8; Supplementary Figures S11 and S12). In addition, we explored the potential correlation of INVS expression with different immunomodulators (Figure 7), MHC molecules, chemokines, and chemokine receptors (Supplementary Figure S13).

#### 2.5.1. INVS and Its Interactome Partners’ Expression in ccRCC Are Correlated with Tumor Leukocyte Infiltration

We assessed the correlation of *INVS* and genes of interest in its interactome (*NPHP3*, *DVL1*, *DVL3*, and *ANKS6*) with tumor immune infiltration and expression using different algorithms (XCELL, TIMER, QUANTISEQ, EPIC, CIBERSORT, CIBERSORT-ABS, and MCPCOUNTER) by using GEPIA [27]. Clinically, the most relevant and statistically significant results according to the TME and INVS levels were the positive correlation observed between the expression of INVS and the infiltration of neutrophils (rho > 0.60 in MPCCOUNTER and QUANTISEQ; *p* < 0.0001), endothelial cells (rho > 0.47 in MPCCOUNTER and EPIC; *p* < 0.0001), and myeloid dendritic cells (rho = 0.43 in MPCCOUNTER; *p* < 0.0001), while the strongest negative correlation was revealed between the expression of INVS and the infiltration of Th1 CD4+ T cells (−0.52 in XCELL; *p* < 0.0001) and T NK cells (−0.47, IN xcell; *p* < 0.0001) (Supplementary Table S3).

#### 2.5.2. INVS and Its Interactome Partners’ Expression in ccRCC Is Correlated with Expression of Immunomodulators

In addition, a significant correlation of the *INVS* expression and its interactome partners with the expression of immune checkpoint genes was found (Figure 8; Supplementary Figures S11 and S12). The expression of *INVS* was correlated with the expression of *CD274* (PD-L1) and *HAVCR* (Figure 8); the expression of *NPHP3* was correlated with the expression of *CTLA4*; the expression of *DVL1* correlated negatively with the expression of *HAVCR2* and *TIGIT* (Supplementary Figure S11); the expression of *DVL3* was correlated with the expression of *CD274 (PD-L1)*, *CTLA4*, *LAG3*, and *PDCD1*; and the expression of *ANKS6* was correlated with the expression of *HAVCR* and negatively correlated with the expression of *TIGIT* (Supplementary Figure S12).

The expression of *INVS* in ccRCC (from the KIRC cohort) is correlated with the immune inhibitory genes *CD274*, *KDR*, LGALS9, and *PVRL2*. In addition, the *INVS* expression in ccRCC is also correlated with the expression of the immune stimulatory genes *IL6R*, *TNFRSF18*, and *TNFSF15* (Figure 7). However, no correlation higher than rho = 0.3 was found between the expression of *INVS* and MHC molecules, chemokines, or chemokine receptors in ccRCC (Supplementary Figure S13).

Despite a correlation with the expression of immune checkpoint genes, when exploring the dataset from Miao et al. (2018) [28], no response in the expression of *INVS* or its interactome partners *NPHP3*, *DVL1*, *DVL3*, and *ANKS6* after treatment with various immune checkpoint inhibitors (pembrolizumab, ipilimumab, nivolumab, and atezolizumab) was observed in ccRCC, urothelial cancer, or melanoma. A response was only observed in urothelial cancer, where the expression of *NPHP3* decreased significantly in response to atezolizumab therapy (Supplementary Table S4).

## 3. Discussion

The aim of this study was to explore the prognostic potential of INVS expression in ccRCC, its relation to metastasis occurrence, and its potential mechanisms in ccRCC pathogenesis, as well as its role as a potential therapeutic target in ccRCC The results of this study show that the INVS expression decreases in ccRCC tissue in comparison to normal renal tissue and that its lower expression is related to the poorer overall and disease-specific survival of patients and a shorter progression-free interval. Moreover, we found that the expression of a distinct group of INVS-interacting factors, including NPHP3, DVL1, DVL3, and ANKS6, is strongly predictive in terms of the overall and/or disease-specific survival length of ccRCC patients, making INVS and this distinguished interactome group potentially clinically useful prognostic indicators.

The tumor differentiation grade is one of the most important factors that determines the stage of disease and the survival of patients [29]. The data obtained from bioinformatics showed a clear relationship between the INVS expression and the histological grade of tumors, where grade 4 ccRCC had the lowest INVS expression, while the expression of INVS was the highest in grade 1 ccRCC. Similar results were also found for ANKS6; on the other hand, the expression of DVL3 was positively related with tumor’s histological grade, being the highest in grade 4 and the lowest in grade 1 ccRCC.

The correlation between a low INVS expression level and poorer patient survival is in agreement with the already-described role of INVS in the pathogenesis of different renal pathologies. Namely, it is known that the consecutive constitutive activation of the canonical Wnt pathway in the absence of INVS leads to abnormal primary cilia and cyst formation, resulting in severe polycystic kidney diseases, MCDK, FSGS, or CNF [15,17]. Hence, the activation of the canonical Wnt pathway might be one of the explanations for the relationship between the poorer survival of patients and lower INVS expression. The involvement of the Wnt signaling pathway in renal carcinoma has previously been studied. The transcriptional coactivator β-catenin, the effector of canonical Wnt signaling, appears to be a key molecule in the pathogenesis of renal cancer [30]. It was found that the overexpression of β-catenin induces renal tumors in mice, while several studies have connected RCC with the hypermethylation or deletion of genes related to the β-catenin and Wnt signaling pathway [30]. The previous studies agree that the Wnt pathway in glioblastoma multiforme (GBM), under the normoxic conditions, promotes cell motility and invasion via an increased epithelial–mesenchymal transformation (EMT), sustains the growth and maintenance of glioma stem cells, and promotes chemo- and radio-resistance [31,32].

Hence, the development of targeted agents might be aimed at the inhibition of the canonical Wnt signaling pathway, especially regarding the inhibition of its effector—transcriptional coactivator B-catenin—which is a key molecule in the pathogenesis of renal cancer. Based on our findings, those patients with a low or absent expression of INVS should experience a positive clinical impact of the inhibition of the canonical Wnt signaling pathway. Therefore, the development of the inhibitor of transcriptional coactivator B-catenin should enable a longer overall survival in those patients.

Metastatic events, including those resulting from ccRCC, are the most important causes of mortality in cancer [33,34,35]. Hence, indicators for a prediction of the metastatic potential are valuable in individualized surveillance protocols and in the prognosis of a relapse. We found that the expression of INVS, as well as ANKS6, one of its interacting partners, was significantly lower in metastatic ccRCC, in comparison to non-metastatic primary ccRCC. It was found that, in cultures of mouse embryonic fibroblasts (MEFs) derived from INVS knockout animals, Wnt signaling is disturbed and INVS is involved in cell polarity and the control of cell migration processes. Therefore, it can be assumed that INVS absence/low expression in ccRCC might have a role in the metastatic potential of tumor cells. On the other hand, the expression of DVL3 was significantly higher in metastatic ccRCC when compared to non-metastatic tumors. This relation to metastatic processes explains the relationship of these factors with the survival rate of ccRCC patients. The results of our study indicate that a distinct group of INVS-interacting factors, including NPHP3, DVL1, DVL3, and ANKS6, are predictive in terms of the overall and disease-specific survival. However, among the studied molecules of interest, only DVL3 expression turned out to be strongly related to patient survival in an isolated group of patients with metastatic ccRCC. This points to its strong prognostic potential, but also its possible predictive role as a therapeutic target, at least for those patients with a high expression level of DVL3. Therefore, the development of therapeutic agents targeted to inhibit DVL3, might allow for a longer overall and/or disease-specific survival in patients who are diagnosed with metastatic ccRCC with a high expression level of DVL3. Whether the mentioned targeted therapeutic options should be applicable alone or together with immunotherapy and/or TKI, the standard treatment in the setting of metastatic ccRCC, acting synergistically, is an unexplored field and needs further preclinical investigations and clinical studies. In order to reach clinical use, these relations need to be further explored. This is not surprising, since many studies have revealed the role of DVL3 in the pathogenesis and prognosis of different types of tumors, including colorectal cancer [36], liver cancer [37], lung adenocarcinoma [38], pancreatic adenocarcinoma [39], and prostate cancer [40]. In support, it has recently been shown that DVL3 increases the proliferation and migration of prostate cancer cells via the Toll-like receptor 4 (TLR4) pathway [40]. However, its role in renal carcinoma has not been studied yet.

According to our results, a distinguished group of patients who might benefit from possible personalized treatment strategies are those with diagnosed ccRCC with a low or absent expression of INVS, as well as those with a high expression of DVL3 in tumor specimens, which can both be established easily with a simple and inexpensive method.

Because the low/absent expression of INVS and the high expression of DVL3 were recorded in tumors that developed metastases, they could be used as additional diagnostic tools in early ccRCC to form an individualized follow-up of the patients and to predict a possible relapse in patients who are at a greater risk, or to involve those uncostly criteria as possible additions, including the risk factors for the treatment with adjuvant immunotherapy.

The investigation of the tumor immune microenvironment (TME) showed that the majority of ccRCC cases are inflammatory neoplasia, which is characterized by a high level of immune cell infiltration, mostly T cells (with 22 different phenotypes composing more than half of the immune cell subset) and tumor-associated macrophages (TAMs), accounting for 31% [41]. Consequently, tumor immune cell infiltrates (lymphocytes, plasma cells, macrophages, neutrophils) have a strong prognostic impact [41,42,43,44,45,46,47,48,49]. In this study, we found multiple correlations of INVS and its interactome with various inflammatory infiltrates in primary ccRCC. Moreover, we also found a correlation with several immunomodulatory molecules, including immunoinhibitors. Clinically, the most relevant results according to the TME and INVS levels are as follows: the strongest positive correlation was observed between the expression of INVS and the infiltration of neutrophils, endothelial cells, and myeloid dendritic cells, while the strongest negative correlation was revealed between the expression of INVS and the infiltration of Th1 CD4+ T cells and T NK cells. Hence, the tumors with a very low INVS expression, which had the worst prognosis, had low neutrophils, low monocytes, and endothelial cell infiltration. On the other hand, in these tumors, higher T cell CD4+ Th1 and T cell NK infiltration is expected. Altogether, these data point to the role of inversin and its interactome partners in the regulation of the tumor microenvironment, with inflammatory infiltration as the main mechanism of their relationship with ccRCC patient survival. These results are in agreement with an immunogenic character of ccRCC, but probably with an affected phenotype and function of effector T cells, which became “exhausted” and were unable to control the cancer growth. Finding that the expression of INVS and its interactome partners was not responsive to therapy with immune checkpoint inhibitors in ccRCC (as well as other types of immune-dependent carcinoma) supports their upstream position in the regulation of the inflammatory tumor microenvironment.

In our study, we compared the expression of INVS between tumors and normal renal tissues by using immunohistochemistry and rtPCR in our cohort, and we also employed a bioinformatics analysis of publicly available data from the TCGA-KIRC cohort. The bioinformatics analysis enabled us to support our data and to more deeply explore the relationship between the INVS expression, its interactome network, and the occurrence of metastases, the degree of differentiation, the expression of immunomodulatory molecules, leukocyte infiltration, and the clinical outcome of patients. The hypotheses were that the expression of INVS is decreased in tumors and that a lower INVS expression is related to the poorer survival of patients with ccRCC. In addition, we hypothesized that the INVS expression might be related to the regulation of tumor leukocyte infiltration, identifying INVS as a potential predictive factor of the response to immunotherapy in ccRCC. Moreover, we hypothesized the existence of the INVS interactome network of factors, which, in cooperation with INVS, affect the biological and clinical characteristics of this type of tumor and have potential as diagnostic and pharmacological targets.

Although the results of our study supported our hypotheses, this study had some limitations, since we did not use a mechanistic approach to prove the role of inversin in ccRCC. Therefore, further studies are needed to possibly introduce the expression of INVS in clinical practice from the perspective of an additional prognostic factor in ccRCC. INVS’s deficiency or minor expression would mean a worse prognosis for patients and indicate the metastatic potential of their ccRCC, but it could also be a predictive factor for immunotherapy, as we proved its connection with the infiltration of different immune cell types in the tumor microenvironment. Some of the genes involved in the INVS interactome network could have potential as pharmacological targets in the future, for example. However, there is a need for further preclinical studies, including overexpressing or silencing INVS and its related genes/proteins in renal cancer cells, applying immunotherapy to establish possible effects depending on the expression of INVS, and performing later clinical studies. However, our study provides a novelty in light of precision oncology, as it is the first one to describe the relationship between the expression of INVS and its interactome partners and some clinical features in patients with ccRCC, serving as an announcement and a basis for future studies.

## 4. Materials and Methods

### 4.1. Patients and Specimens

The ethical approval for this study and the specimens were obtained from the Clinical Hospital Mostar. The study was approved by the institutional review board (or ethics committee) of the Clinical Hospital Mostar (protocol code 01-I-245/21 and 141/24). Primary tumor specimens were obtained from 34 patients who were diagnosed with ccRCC, and all of them underwent a radical nephrectomy between 2009 and 2019. All of the patients eventually developed metastatic disease, either synchronous (21 of them) or metachronous (diagnosed in 13 patients during follow-up). Among the patients, only 6 of them developed metastasis one year after the initial diagnosis of ccRCC. The clinical data were obtained from the hospital case records and the patients were identified from the hospital registry. None of the patients had received prior therapy before their surgical resection. The mean age of the patients was 60.3 (range: 46–76) and this did not differ between the sexes, with a mean age of 60.7 (range: 49–76) at the time of diagnosis for females and 59.9 (range: 46–76) for males (Supplementary Table S2). The histological diagnosis and grading were evaluated using hematoxylin-and-eosin-stained sections. According to the grade, a total of 14 tumors were classified as grade 2, 14 as grade 3, and 6 tumor samples were classified as poorly differentiated grade 4. Grade 1 was not represented in these samples.

### 4.2. Immunohistochemistry, Data Acquisition, and Immunohistochemical Analysis

The surgically excised primary tumor and surrounding normal kidney tissue specimens obtained from the radical nephrectomy were fixed with 10% neutral-buffered formalin, embedded in paraffin, cut into 2-µm-thick sections, and mounted on histological slides. The sections were deparaffinized using xylene and rehydrated with graded ethanol and water. After deparaffinization, the sections were heated for 30 min in citrate buffer (pH of 6.0), cooled down, and washed in phosphate-buffered saline (PBS). The sections were then blocked with a protein-blocking buffer (ab64226, Abcam, Cambridge, UK), and a primary antibody—rabbit polyclonal anti-inversin antibody (dilution of 1:50; ab65187, Abcam, Cambridge, UK)—was applied overnight at room temperature in a humid chamber. The inversin antibody has previously been validated in our laboratory and has been used for immunofluorescence on mice and human tissues in previous studies [17,50]. In addition, the omission of the primary antibody from the procedure resulted in no staining in the tissue. Normal renal tissue was used as a positive control [51]. After washing in PBS, followed by a secondary antibody incubation (donkey anti-rabbit lgG, Alexa Fluor^®^488, 711-545-152, Jackson Immuno Research Laboratories, Inc., Baltimore, PA, USA; diluted at 1:300) at room temperature for 1 h, the sections were washed in PBS. The slides were then air-dried and cover-slipped (ImmuMount, Shandon, Pittsburgh, PA, USA).

The sections were observed under a fluorescence microscope (Olympus BX61, Tokyo, Japan). Ten visual fields were captured using a cooled digital DS-Ri2 digital camera (Nikon, Tokyo, Japan) equipped with the NIS-Elements F software version 4.60 at an objective magnification of ×40. The photomicrographs were processed using the ImageJ software (version 1.48; National Institutes of Health, Bethesda, MD, USA). Initially, the red counter-signal was subtracted and the median filter with a radius of 20 pixels was applied. Subsequently, the triangle threshold algorithm was applied and the positive percentage area was measured using the “analyze particles” function, without the limitation of particle size or circularity of the analyzed particles. Mean value of the percentage area of all photomicrographs of one specimen was used as a percentage area measure. We analysed the entire area of the tumor and/or renal tissue. However, the highest expression of INVS was observed in the tubular cells, while very low expression of INVS was found in ccRCC tumor tissue. The expression pattern in the normal tissue was cytoplasmic and/or nuclear. In the tumor specimens, a weak, mostly nuclear expression was found. The figures for illustration were prepared in Adobe Photoshop (Adobe Inc., San Jose, CA, USA). The subtraction of the background and slight contrasting were applied for this purpose. The GraphPad Prism 8 software (version 8.0.1 for Windows, GraphPad Software, San Diego, CA, USA) and an unpaired *t*-test were used for a comparison of two groups. Statistical significance was considered at *p* < 0.05.

### 4.3. RNA Isolation and RT-rtPCR

Twenty-eight primary ccRCC samples and 10 normal border renal tissue samples were used for the isolation of RNA. RNA was isolated using the GenEluteTM FFPE RNA Purification Kit (Sigma-Aldrich, Taufkirchen, Germany), as previously reported [52]. The QubitTM 4 Fluorometer (Thermo Fisher Scientific Inc., Waltham, MA, USA)was used to measure the concentration of RNA. All the samples were diluted to match the same concentration (5 ng/μL). The one-step iTaq™ Universal SYBR^®^ Green One-Step Kit designed for measuring the gene expression was used as previously reported [53]. The primers used in the RT-qPCR method (Supplementary Table S1) were designed using the Primer-BLAST software (it uses Primer3 version 2.5.0 to design PCR primers and then uses BLAST and global alignment algorithm to screen primers against user-selected database in order to avoid primer pairs that can cause non-specific amplifications; NCBI, Bethesda, MD, USA). RPL 13a was used as the reference gene. The Applied Biosystems™ 7500 RT-PCR system (Thermo Fisher Scientific, Waltham, MA, USA) was used for the analysis. A negative control using nuclease-free water instead of a cDNA template was included in each experiment. A normal renal tissue sample was used as a positive control [54]. The 2^−ΔΔCt^ method was used as the method of relative quantification [28,55]. An unpaired *t*-test was used for comparisons of two groups.

### 4.4. Normal INVS Expression in Kidneys and INVS Interactome Network

Data from the Human Protein Atlas (https://www.proteinatlas.org/) (accessed on 1 March 2024) [51,54,56,57] were used to confirm the expression of INVS mRNA and protein in different tissues, including the kidneys, as well as the INVS mRNA presence in different types of tumors, including ccRCC, and in various cell lines of normal renal cells and renal tumor lines. We used the data on a single-cell transcriptome analysis of the kidneys in the HPA from the GSE131685 dataset [21] to confirm the INVS mRNA expression in different clusters of cells in normal human kidneys. The STRING database [24] (12.0 version) (accessed on 23 September 2023, with the query “INVS” and the species *Homo sapiens*) was used to determine the INVS interactome network. As a result, 10 (partners) were identified.

### 4.5. Survival and Statistical Analysis in the TCGA-KIRC Cohort

The RNA-seq and clinical data of ccRCC (TCGA-KIRC data) from the TCGA data portal were downloaded from the University of California Santa Cruz (UCSC) Xena database [25] (https://xenabrowser.net/datapages/) (accessed on 10 May 2024). Samples with no expression data for INVS were removed by filtering, and 605 matching samples were left. We used data only for the expression in the primary tumor (N = 533) and a normal solid tissue specimen (N = 72). Data from the primary tumor (533 samples) were used for a Kaplan–Meyer curve analysis. The median was used as the cut-off value. The different Kaplan–Meier curves were compared using the log-rank test and *χ*2, and the *p*-values (*χ*2 distribution) were reported. A differential expression analysis using Welch’s *t*-test was made to compare the M1 (N = 79) vs. M0 (N = 422) pathological M stages (for data with a known M stage). A one-way ANOVA was used to compare expression data according to a histological grade, after GX (N = 5) and null (N = 3) specimens were removed. N = 525 (14—G1, 229—G2, 206—G3, 76—G4).

### 4.6. The Association Between INVS, Its Interactome, and the Immune Microenvironment

In order to reveal the mechanism of INVS and its interactome effects on ccRCC pathogenesis and the clinical outcome, we used TISIDB [26] (http://cis.hku.hk/TISIDB/index.php) (accessed on 1 March 2024) to explore the correlation of the expression of INVS and its partners with tumor leukocyte infiltration in ccRCC (from the KIRC cohort). We assessed the correlation of *INVS* and genes of interest in its interactome (*NPHP3*, *DVL1*, *DVL3*, and *ANKS6*) with the tumor immune infiltration and expression using different algorithms (XCELL, TIMER, QUANTISEQ, EPIC, CIBERSORT, CIBERSORT-ABS, and MCPCOUNTER). We used the GEPIA [27] (http://gepia.cancer-pku.cn/) (accessed on 9 February 2024) database to explore the correlation of the expression of *INVS* and its partners with the immune checkpoint genes in ccRCC (from the KIRC cohort) [49,50]. In addition, we used TIMER 2.0 (http://timer.cistrome.org/) (accessed on 29 January and 9 February 2024) to explore the potential correlation of the *INVS* expression in ccRCC (from the KIRC cohort) with different immunomodulators, MHC molecules, chemokines, and chemokine receptors [58]. In addition, we explored the dataset from Miao et al. (2018) [28] to investigate the potential response in the expression of *INVS* and its interactome partners after treatment with various immune checkpoint inhibitors (pembrolizumab, ipilimumab, nivolumab, and atezolizumab) in ccRCC, non-small cell lung cancer, urothelial cancer, or melanoma.

## 5. Conclusions

The results of this study point to INVS and a distinguished group of its interactome partners as potential prognostic factors in ccRCC, with their predominant involvement in the modulation of the inflammatory infiltration of the tumor microenvironment and a strong relationship with the metastatic potential of the tumor. Whether the INVS interactome partners could also have potential as therapeutic targets, especially for an individual therapeutic approach, deserves to be further explored.

## Data Availability

The datasets presented in this study can be found in online repositories. The names of the repository/repositories and accession number(s) can be found below: https://docs.gdc.cancer.gov/Data/Release_Notes/Data_Release_Notes/#data-release-400; https://gdc-hub.s3.us-east-1.amazonaws.com/download/TCGA-KIRC.star_fpkm-uq.tsv.gz; Full metadata, TCGA_KIRC_exp_HiSeqV2; https://www.ncbi.nlm.nih.gov/geo/query/acc.cgi?acc=GSE131685, GSE131685.

## Supplementary Materials

The supporting information can be downloaded at: https://www.mdpi.com/article/10.3390/ijms252212120/s1.
