# Supplementary material for: Identifying an Inversin as a Novel Prognostic Marker in Patients with Clear-Cell Renal Cell Carcinoma
Source: Int J Mol Sci. 2024 Nov 12;25(22):12120. doi: 10.3390/ijms252212120 (PMC11594840; doi:10.3390/ijms252212120)
Supplement: Supplementary file 1 [file ijms-25-12120-s001.zip › ijms-3290152-supplementary.pdf]

Supplementary Table S1. Primers used in RT-rtPCR.

Transcript	Forward Primer	Reverse Primer	T_m F/R	CG% F/R
RPL13a	CCTGGAGGAGAAGAGGAAAGAGA	TTGAGGACCTCTGTGTATTTGTCAA	63.1/60.5	52.17/40.00
INVS*	TGCACTATGCTGCTCAGAGTAA	GCCAGCTGCCCACATAAAGG	59.50/61.67	45.45/60.00

*INVS primers are designed to recognize 3 variants: *Homo sapiens* inversin (INVS), transcript variant 1, *Homo sapiens* inversin (INVS), transcript variant 4 and *Homo sapiens* inversin (INVS), transcript variant 5

Supplementary Table S2. Clinical and pathological characteristics of ccRCC patients.

	**No. (%)**
**Characteristics**	
No. of patients	34
Age. years	
Mean (range)	60.7 (46-76)
Gender	
Female	10 (29)
Male	24 (70.5)
ECOG PS	
0	16 (47)
1	13 (38)
2	5 (14.7)
3	0
4	0
Histological grade	
1	0
2	14 (41)
3	14 (41)
4	6 (17.6)
TNM disease stage at initial diagnosis	
I	9 (26)
II	2 (5.9)
III	19 (55.8)
IV	4 (11.7)
Metastatic disease. months	
<12	28 (82)
≥12	6 (17.6)
Disease site	
lungs	25 (73.5)
liver	5 (14.7)
lymph nodes	4 (11.7)
bone	10 (29)
bone only	3 (8.8)
brain	1 (2.9)
No of disease sites	
1	21 (61.7)
2	10 (29)
3	2 (5.8)
4	1 (2.9)
IMDC prognostic group	
favorable	3 (8.8)
intermediate	15 (44)
poor	16 (47)
Median OS	
Months (range)	32.6 (2-118)

Supplementary Table S3. Correlation analysis of INVS and inversin-interactome partners expression and abundance of tumor-infiltrating lymphocytes in TCGA-KIRC, using different algorithms.

infiltrates	INVS-rho	INVS-adj.p	NPHP3-rho	NPHP3-adj.p	DVL1-rho	DVL1-adj.p	DVL3-rho	DVL3-adj.p	ANKS6-rho	ANKS6-adj.p
**T cell CD8+ central memory_XCELL**	**-0,136306831**	**0,023534078**	-0,09154704	0,151099369	**-0,164448171**	**0,003074272**	**-0,154133456**	**0,006423763**	**-0,240457396**	**7,21E-06**
T cell CD8+ effector memory_XCELL	**-0,17202181**	**0,002751062**	0,024557386	0,744031904	0,018839852	0,806481424	-0,086965013	0,160220629	**-0,190528034**	**0,00072959**
T cell CD8+ naive_XCELL	0,016108996	0,877165768	**0,25170366**	**2,15E-06**	**0,151099418**	**0,006690441**	0,017494491	0,818421393	**0,068781623**	0,324890684
T cell CD8+_CIBERSORT	**-0,16676423**	**0,004038239**	-0,054222552	0,443937304	0,068258202	0,291141027	-0,100172084	0,097757858	-0,06913568	0,323509755
T cell CD8+_CIBERSORT-ABS	0,040534202	0,626394726	0,079889148	0,232600024	0,065837241	0,316324535	0,066825745	0,305988498	-0,018963728	0,855836769
T cell CD8+_EPIC	**0,328575598**	**3,65E-11**	**0,119020115**	**0,046324532**	**-0,161569519**	**0,00348556**	0,070574219	0,274979306	**0,125237418**	**0,040553025**
T cell CD8+_MCPCOUNTER	-0,01423686	0,892046689	-0,009087839	0,914278182	-0,047774796	0,500330208	-0,034212825	0,643999951	-0,085350595	0,208099812
T cell CD8+_QUANTISEQ	**-0,136101004**	**0,023534078**	-0,019411384	0,803571681	-0,099627681	0,096916531	-0,058987292	0,385360341	**-0,180277091**	**0,001472063**
T cell CD8+_TIMER	0,03314822	0,697100046	0,014440914	0,843493919	0,036849479	0,620837091	-0,087959526	0,154631351	-0,073306493	0,282919707
T cell CD8+_XCELL	-0,085656131	0,194075654	0,04439003	0,539286226	**-0,114929297**	**0,049704331**	-0,053738663	0,433946608	**-0,167977795**	**0,003763061**
T cell CD4+ (non-regulatory)_QUANTISEQ	**0,354577432**	**1,38E-13**	0,044334008	0,512044112	0,088629396	0,170617624	0,059873777	0,387080527	**0,342686833**	**5,98E-12**
T cell CD4+ (non-regulatory)_XCELL	0,04362835	0,52676539	0,028152123	0,70436957	-0,028962162	0,748150397	0,001776067	0,984619793	0,05901629	0,477114762
T cell CD4+ central memory_XCELL	0,07366044	0,23750359	**0,355525231**	**1,26E-13**	**0,289855524**	**1,05E-08**	**0,205762808**	**8,03E-05**	**0,176331897**	**0,002191364**
T cell CD4+ effector memory_XCELL	**-0,249301947**	**6,97E-07**	-0,095642205	0,101876435	-0,068584142	0,328229421	**-0,209008617**	**5,95E-05**	**-0,215330742**	**8,10E-05**
T cell CD4+ memory activated_CIBERSORT	-0,025660879	0,737615865	0,044460033	0,510959439	-0,026174205	0,780624754	0,037107195	0,624189808	**-0,135703615**	**0,028573164**
T cell CD4+ memory activated_CIBERSORT-ABS	-0,024622163	0,746896514	0,045608495	0,499863419	-0,026025173	0,780624754	0,038055387	0,615073924	**-0,13588254**	**0,028512981**
T cell CD4+ memory resting_CIBERSORT	**0,132386214**	**0,017417477**	0,050660763	0,44143126	-0,06188	0,391187087	0,031826374	0,68548032	0,023314396	0,829735092

					645					
T cell CD4+ memory resting_CIBERSORT-ABS	**0,318750261**	**5,58E-11**	**0,199054272**	**0,0001102**	-0,017710628	0,868253498	**0,175913015**	**0,001026373**	0,083329813	0,250459623
T cell CD4+ memory_XCELL	-0,065198556	0,308852841	-0,012101892	0,885755019	-0,179890233	**0,001003601**	-0,110019128	0,065099218	-0,170145144	**0,003421061**
T cell CD4+ naive_CIBERSORT	0,011050649	0,900331115	0,06767784	0,279505753	0,057491916	0,426135138	0,06147273	0,380464851	0,068560883	0,381832791
T cell CD4+ naive_CIBERSORT-ABS	0,011050649	0,900331115	0,06767784	0,279505753	0,057491916	0,426135138	0,06147273	0,380464851	0,068560883	0,381832791
T cell CD4+ naive_XCELL	-0,023692717	0,753747903	**0,229710746**	**5,95E-06**	0,080359125	0,232475801	**0,185841582**	**0,000475617**	-0,064622874	0,413674392
T cell CD4+ Th1_XCELL	**-0,520464179**	**4,57E-31**	**-0,218593587**	**1,83E-05**	0,110148727	0,070716211	**-0,219167379**	**2,39E-05**	**-0,263920817**	**4,06E-07**
T cell CD4+ Th2_XCELL	**-0,170891721**	**0,001371683**	**-0,184813943**	**0,000383745**	**-0,381962663**	**1,82E-15**	-0,11547898	0,052271552	**-0,327310882**	**6,40E-11**
**T cell CD4+_EPIC**	**0,499030998**	**2,67E-28**	**0,166287702**	**0,001670625**	**-0,147506422**	**0,009672866**	**0,283431281**	**1,59E-08**	**0,292308795**	**1,12E-08**
**T cell CD4+_TIMER**	**0,296413431**	**1,62E-09**	**0,440955591**	**2,66E-21**	**0,239328048**	**5,07E-06**	**0,435056649**	**2,04E-20**	**0,181567498**	**0,001517774**
T cell regulatory (Tregs)_CIBERSORT	**-0,362047611**	**3,45E-14**	**-0,136251692**	**0,013205904**	0,058953953	0,412849493	-0,064277096	0,35310607	**-0,196393583**	**0,000450954**
T cell regulatory (Tregs)_CIBERSORT-ABS	**-0,288266606**	**5,11E-09**	-0,081870765	0,17799894	0,045412048	0,552886074	0,007876349	0,927091035	**-0,169022661**	**0,003699051**
T cell regulatory (Tregs)_QUANTISEQ	0,069736548	0,264856403	**0,22288328**	**1,16E-05**	0,010339309	0,932158011	**0,216286778**	**3,18E-05**	-0,110603119	0,099577944
T cell regulatory (Tregs)_XCELL	0,04672253	0,495631201	0,056306194	0,387471704	-0,062812577	0,382034326	0,010267199	0,908813916	0,002399341	0,987626303
B cell memory_CIBERSORT	**-0,246861915**	**2,16E-06**	**-0,132826579**	**0,025932745**	-0,006343153	0,962546454	**-0,129001712**	**0,030036564**	**-0,180788301**	**0,001962744**
B cell memory_CIBERSORT-ABS	**-0,240337787**	**4,59E-06**	**-0,130076447**	**0,028907843**	-0,010641974	0,940428664	**-0,125262298**	**0,035317526**	**-0,181884851**	**0,001839761**
B cell memory_XCELL	**-0,137533444**	**0,017014193**	-0,087999651	0,169465178	-0,045954515	0,564967982	-0,012827882	0,873043306	-0,078342479	0,283059047
**B cell naive_CIBERSORT**	**0,269618477**	**1,62E-07**	**0,137143204**	**0,021147361**	**-0,126113833**	**0,036896131**	**0,179041278**	**0,001257525**	**0,12565962**	**0,046498976**
B cell naive_CIBERSORT-ABS	**0,317357436**	**3,63E-10**	**0,187583394**	**0,000723339**	-0,10147	0,110840599	**0,216514082**	**6,25E-05**	**0,151158665**	**0,013064049**

					3872					
B cell naive_XCELL	**-0,1208904**	**0,040462035**	-0,052151529	0,457451606	**-0,170156465**	**0,002719807**	0,090193814	0,149188381	-0,111567842	0,079757158
B cell plasma_CIBERSORT	0,054874724	0,42288659	0,025837946	0,722020868	0,039313454	0,642885618	0,00175012	0,989215028	0,000649187	0,992216509
B cell plasma_CIBERSORT-ABS	**0,248018198**	**1,96E-06**	**0,158434567**	**0,006294709**	0,026910573	0,776393701	**0,146391039**	**0,012182148**	0,057509806	0,465299206
B cell plasma_XCELL	**-0,227985753**	**1,72E-05**	-0,102251282	0,102978681	**-0,148442111**	**0,010441633**	-0,098498452	0,111521463	**-0,220027687**	**6,91E-05**
B cell_EPIC	**0,16328164**	**0,004177233**	-0,001296056	0,982773846	-0,058393195	0,442104296	0,039881009	0,564029533	0,049742078	0,55636604
B cell_MCPCOUNTER	**0,119509857**	**0,042889281**	0,067273803	0,31870928	**-0,167993029**	**0,003070202**	0,109500519	0,069631266	-0,086587623	0,217295928
B cell_QUANTISEQ	**0,207367855**	**0,000115944**	**0,253921402**	**1,41E-06**	**-0,162874871**	**0,004390231**	**0,221499478**	**3,76E-05**	-0,04666976	0,58955112
B cell_TIMER	-0,039530258	0,583299772	**-0,150017462**	**0,010154376**	0,002675478	0,982142277	**-0,132597072**	**0,025477831**	0,014703657	0,907114927
B cell_XCELL	**-0,183796168**	**0,000847611**	-0,07659313	0,252281208	**-0,280918032**	**7,11E-08**	-0,007058723	0,932902896	**-0,262559604**	**1,26E-06**
Class-switched memory B cell_XCELL	-0,111045677	0,064120119	-0,038989341	0,577084815	-0,110537359	0,079635416	-0,02541739	0,735824613	-0,121169598	0,053650324
Cancer associated fibroblast_EPIC	-0,002275426	0,976469315	0,06706702	0,267823421	-0,06574159	0,361044557	**0,226478621**	**6,19E-06**	-0,089237082	0,198332909
Cancer associated fibroblast_MCPCOUNTER	0,026384766	0,681089126	**0,127011675**	**0,02193831**	0,070300198	0,325585006	**0,277788216**	**1,42E-08**	-0,02123662	0,839924581
Cancer associated fibroblast_TIDE	0,070913232	0,214113428	0,054465678	0,386270111	-0,010564552	0,900164884	**0,231343238**	**3,66E-06**	-0,004745234	0,954666134
Cancer associated fibroblast_XCELL	-0,108520692	0,04580497	-0,076791049	0,203377772	-0,074072043	0,288262539	0,048081939	0,451921967	-0,008920034	0,924639552
Macrophage/Monocyte_MCPCOUNTER	**0,366939735**	**1,54E-14**	**0,262028543**	**1,84E-07**	0,057397195	0,438053032	**0,269096545**	**4,01E-08**	**0,138709953**	**0,020555161**
**Monocyte_CIBERSORT**	**0,20652227**	**4,36E-05**	**0,176431607**	**0,000865799**	**0,201237298**	**0,000208033**	**0,211991977**	**2,72E-05**	**0,150965449**	**0,010027226**
**Monocyte_CIBERSORT-ABS**	**0,325990546**	**1,51E-11**	**0,266568202**	**1,15E-07**	**0,209343727**	**0,000105876**	**0,304704297**	**4,00E-10**	**0,19827554**	**0,000306756**
Monocyte_MCPCOUNTER	**0,366939735**	**1,54E-14**	**0,262028543**	**1,84E-07**	0,057397195	0,438053032	**0,269096545**	**4,01E-08**	**0,138709953**	**0,020555161**
Monocyte_QUANTISEQ	**-0,22649**	**6,47E-06**	-0,04112	0,526073996	0,040732698	0,611304749	-0,00339	0,961601136	-0,03638	0,702763734

	**2319**		7476				9188		4222	
Monocyte_XCELL	-0,024760024	0,701680676	-0,003109115	0,965294355	**-0,151767685**	**0,009427927**	0,014454544	0,835630915	**-0,203251375**	**0,000193197**
Neutrophil_CIBERSORT	**0,1263521**	**0,018119166**	0,026404508	0,692416142	**-0,130203128**	**0,03192218**	0,082978397	0,164960243	-0,0884282	0,203424342
Neutrophil_CIBERSORT-ABS	**0,156326517**	**0,002922128**	0,054880262	0,381544271	-0,118366987	0,060791815	**0,117896131**	**0,034111936**	-0,067932453	0,36157521
**Neutrophil_MCPCOUNTER**	**0,636065104**	**7,80E-51**	**0,303946699**	**7,94E-10**	**0,231268164**	**1,43E-05**	**0,318560119**	**4,51E-11**	**0,456524797**	**4,36E-23**
**Neutrophil_QUANTISEQ**	**0,604757184**	**8,17E-45**	**0,172049468**	**0,00119198**	0,114312897	0,069415167	**0,165333076**	**0,001588862**	**0,323526545**	**5,73E-11**
Neutrophil_TIMER	**0,356461487**	**9,85E-14**	**0,282106062**	**1,71E-08**	-0,059508549	0,420199754	**0,292902345**	**2,02E-09**	0,011473228	0,912753848
Neutrophil_XCELL	**0,138150456**	**0,009271155**	**0,023522506**	0,723469917	0,022340079	0,801932506	0,067735303	0,26726045	0,048133649	0,571001734
Macrophage M0_CIBERSORT	**-0,230382548**	**5,05E-06**	**-0,137437643**	**0,011950185**	-0,08484369	0,151671432	0,020761205	0,762022121	**-0,194149794**	**0,00031784**
Macrophage M0_CIBERSORT-ABS	**-0,219619504**	**1,56E-05**	**-0,131368905**	**0,01707841**	-0,088132258	0,136921288	0,024871715	0,723129936	**-0,191660681**	**0,000390332**
Macrophage M1_CIBERSORT	**0,196978449**	**0,000144541**	0,10391148	0,069711792	-0,069433631	0,25142052	0,019156664	0,780498961	0,013565451	0,895358218
Macrophage M1_CIBERSORT-ABS	**0,343110794**	**1,75E-12**	**0,214843023**	**2,99E-05**	-0,05092071	0,414505149	**0,166196747**	**0,001831415**	0,047447436	0,508552137
Macrophage M1_QUANTISEQ	**-0,127617333**	**0,020018744**	0,008837768	0,914133504	-0,031676015	0,637813892	0,055560608	0,377102547	**-0,186292052**	**0,000612313**
**Macrophage M1_XCELL**	**-0,242111553**	**1,52E-06**	**-0,290449496**	**4,41E-09**	**-0,320890377**	**1,26E-10**	**-0,151268816**	**0,004953027**	**-0,302716066**	**2,38E-09**
Macrophage M2_CIBERSORT	**0,147738809**	**0,006074248**	-0,054390682	0,368482613	**-0,115808199**	**0,043286104**	0,037111351	0,571191921	**0,120333092**	**0,039897083**
Macrophage M2_CIBERSORT-ABS	**0,368971723**	**2,21E-14**	**0,190885495**	**0,000258257**	-0,064532326	0,2878935	**0,236689608**	**2,68E-06**	**0,154349211**	**0,005644401**
Macrophage M2_QUANTISEQ	**0,109918273**	**0,04973395**	0,007698123	0,917888165	**-0,239609029**	**4,44E-06**	0,057136696	0,362507141	-0,061910979	0,374038002
Macrophage M2_TIDE	-0,050510328	0,427363151	**-0,154123905**	**0,00421874**	0,089181109	0,132082818	**-0,152547488**	**0,004624202**	**0,179497381**	**0,001015365**
**Macrophage M2_XCELL**	**-0,16652**	**0,001654854**	**-0,36931**	**2,07E-14**	**-0,24171**	**3,72E-06**	**-0,21425**	**2,73E-05**	**-0,16631**	**0,002425**

	468		591		8133		5992		5462	
Macrophage_EPIC	**-0,136792756**	**0,011822352**	**-0,150741527**	**0,005273651**	**-0,185632977**	**0,000578783**	-0,043974193	0,497299796	**-0,204676063**	**0,000145222**
Macrophage_TIMER	**0,309608147**	**2,67E-10**	0,102658665	0,073722744	**-0,182931537**	**0,000709199**	**0,239910515**	**1,95E-06**	0,115559982	0,049821844
**Macrophage_XCELL**	**-0,175245768**	**0,000826796**	**-0,307842581**	**3,69E-10**	**-0,352082262**	**1,01E-12**	**-0,151584768**	**0,004870024**	**-0,244842041**	**2,65E-06**
Macrophage/Monocyte_MCPCOUNTER	**0,366939735**	**2,89E-14**	**0,262028543**	**1,73E-07**	0,057397195	0,353084147	**0,269096545**	**6,35E-08**	**0,138709953**	**0,014438875**
Myeloid dendritic cell activated_CIBERSORT	-0,016855717	0,845281742	-0,006116951	0,960480615	0,065876323	0,371678534	-0,025406434	0,768957967	0,029063887	0,736827953
Myeloid dendritic cell activated_CIBERSORT-ABS	-0,012247949	0,891122331	-0,002802304	0,984124647	0,067773214	0,351217365	-0,023010985	0,790130024	0,034944376	0,66414435
Myeloid dendritic cell activated_XCELL	-0,051878924	0,441586363	-0,008633161	0,938278935	-0,00724455	0,966497534	0,018133032	0,84875764	**-0,147670882**	**0,013242198**
Myeloid dendritic cell resting_CIBERSORT	0,088108906	0,162144141	-0,0068556	0,951636631	-0,102110011	0,108337177	0,011009561	0,912786823	0,083510841	0,213773123
Myeloid dendritic cell resting_CIBERSORT-ABS	0,093677523	0,135653302	0,003339801	0,984124647	-0,100610473	0,112392203	0,021241771	0,814887332	0,082173606	0,224855471
Myeloid dendritic cell_MCPCOUNTER	**0,433694934**	**1,04E-19**	**0,247909722**	**1,25E-06**	-0,045163499	0,586477295	**0,182096493**	**0,000755802**	0,104249174	0,105188024
Myeloid dendritic cell_QUANTISEQ	**0,22510836**	**2,27E-05**	**0,216565197**	**3,28E-05**	**0,149816188**	**0,00948161**	0,094070511	0,131817073	**0,253699858**	**1,04E-06**
Myeloid dendritic cell_TIMER	0,067280685	0,303513604	**-0,123721845**	**0,035615837**	-0,100811354	0,112392203	0,066612085	0,321868248	-0,080072882	0,240976387
Myeloid dendritic cell_XCELL	-0,045886951	0,518311919	**-0,141868553**	**0,012221612**	**-0,320301412**	**1,67E-10**	-0,088781017	0,15704504	**-0,183737849**	**0,001106566**
NK cell activated_CIBERSORT	**-0,20856767**	**9,25E-05**	-0,026249845	0,773226604	**0,169762071**	**0,002534076**	-0,114188863	0,054378513	-0,000618758	0,990808917
NK cell activated_CIBERSORT-ABS	0,024482831	0,744110763	0,111720072	0,066180606	**0,139444016**	**0,017752825**	0,041508346	0,579832764	0,02665182	0,764162808
NK cell resting_CIBERSORT	0,114936584	0,058610968	**0,138392488**	**0,015333159**	**0,152493097**	**0,008060644**	0,091520854	0,143097895	**0,158695666**	**0,006533925**
NK cell resting_CIBERSORT-ABS	**0,134858538**	**0,021200846**	**0,152899273**	**0,006127643**	**0,157181716**	**0,005836971**	0,108202326	0,07380267	**0,169978677**	**0,003045134**
NK cell_EPIC	**-0,318731582**	**1,92E-10**	-0,006947299	0,951636631	**0,271501675**	**1,17E-07**	**-0,158706691**	**0,004162747**	**-0,173566944**	**0,002313045**
NK cell_MCPCOUNTER	**0,21584**	**4,64E-**	**0,37021**	**2,41E-**	0,12371	0,0401	**0,12176**	**0,0360**	0,07421	0,2860

	**7459**	**05**	**6308**	**14**	7903	52623	**9349**	**563**	4196	16862
**NK cell_QUANTISEQ**	**0,275503542**	**7,55E-08**	**0,334792238**	**8,55E-12**	**0,312836207**	**5,06E-10**	**0,204507773**	**0,000123133**	**0,297480397**	**6,40E-09**
NK cell_XCELL	0,051879066	0,441586363	0,033656795	0,688725261	0,022147952	0,824834294	-0,040081296	0,593848361	0,063264306	0,377532116
**Plasmacytoid dendritic cell_XCELL**	**-0,310248225**	**6,91E-10**	**-0,156575359**	**0,004927383**	**-0,184785491**	**0,000813939**	**-0,179802592**	**0,000897227**	**-0,308599029**	**1,50E-09**
Common lymphoid progenitor_XCELL	0,082784182	0,21826506	-0,050595971	0,500987422	**-0,337233465**	**9,96E-12**	**-0,214564883**	**4,49E-05**	-0,031683505	0,722817136
Common myeloid progenitor_XCELL	0,049912243	0,534822604	0,017302373	0,840553217	0,068780241	0,327882999	0,057930884	0,388611054	0,047941903	0,555401297
Endothelial cell_EPIC	**0,471361882**	**1,87E-24**	**0,212779735**	**5,34E-05**	**0,222254377**	**2,48E-05**	0,109885878	0,068254819	**0,307176881**	**2,88E-09**
**Endothelial cell_MCPCOUNTER**	**0,487676943**	**2,56E-26**	**0,303281718**	**1,15E-09**	**0,246581904**	**1,86E-06**	**0,156968199**	**0,005640994**	**0,305614967**	**2,88E-09**
Endothelial cell_XCELL	**0,312958915**	**2,59E-10**	**0,226904843**	**1,35E-05**	**0,269168473**	**1,77E-07**	0,062980613	0,347932748	**0,217282956**	**6,80E-05**
Eosinophil_CIBERSORT	-0,044995564	0,593828533	0,011792008	0,915001609	**-0,173171102**	**0,001803622**	-0,098812365	0,10660485	-0,124239121	0,056565955
Eosinophil_CIBERSORT-ABS	-0,044987396	0,593828533	0,012481549	0,904620933	**-0,17329205**	**0,001803622**	-0,098424538	0,10755655	-0,123865763	0,056870583
Eosinophil_XCELL	0,0326275	0,732998	-0,02958407	0,693618503	-0,040555009	0,594364119	0,05194644	0,455455288	-0,014768441	0,886066593
Granulocyte-monocyte progenitor_XCELL	**0,297681722**	**2,28E-09**	**0,16210379**	**0,003332547**	-0,050119742	0,501211907	**0,145709428**	**0,011368583**	0,115925985	0,080782953
Hematopoietic stem cell_XCELL	**0,28327433**	**1,51E-08**	0,104807355	0,087132499	**0,127866828**	**0,03196387**	0,038225156	0,614353292	**0,209661315**	**0,000127323**
Mast cell activated_CIBERSORT	**0,142950176**	**0,012749268**	0,046713807	0,534040538	0,098389545	0,129005458	0,048101728	0,501606938	0,111942811	0,098648792
Mast cell activated_CIBERSORT-ABS	**0,226831731**	**1,28E-05**	0,116872359	0,050161552	0,115800116	0,060072992	**0,125182363**	**0,03398065**	**0,156124307**	**0,00857936**
Mast cell resting_CIBERSORT	-0,029224674	0,750174538	0,01013403	0,923859092	-0,047533952	0,532291071	-0,017283699	0,850975741	-0,043466733	0,599008919
Mast cell resting_CIBERSORT-ABS	-0,015676075	0,885871011	0,022299806	0,779640601	-0,048802556	0,515915482	-0,00519115	0,961597662	-0,044411311	0,599008919
Mast cell_XCELL	**0,335074931**	**6,87E-12**	**0,15872324**	**0,004197066**	-0,070949668	0,30588802	**0,185429872**	**0,000645393**	0,014427842	0,887594982
T cell follicular helper_CIBERSORT	**-0,24505574**	**1,82E-06**	0,080806162	0,216436699	**0,135570289**	**0,021573301**	0,007163073	0,939773257	**-0,16140115**	**0,006646968**

T cell follicular helper_CIBERSORT-ABS	-0,107747557	0,080261253	**0,151715283**	**0,006818777**	**0,123827284**	**0,039724208**	0,088995203	0,152930671	-0,101226378	0,141194024
**T cell gamma delta_CIBERSORT**	**-0,135397674**	**0,018922714**	**-0,17414938**	**0,001444558**	**-0,263713392**	**2,96E-07**	**-0,284779559**	**1,88E-08**	**-0,2013497**	**0,000267244**
T cell gamma delta_CIBERSORT-ABS	-0,084738073	0,202707118	**-0,133293108**	**0,021559934**	**-0,255338493**	**6,86E-07**	**-0,235337793**	**6,19E-06**	**-0,175036744**	**0,002327877**
T cell gamma delta_XCELL	-0,020313699	0,840527666	-0,011210988	0,918184613	0,026025455	0,753217197	0,033334712	0,676270485	0,015964927	0,882949437
MDSC_TIDE	**-0,166059135**	**0,001106499**	0,030714247	0,618968314	**0,195495773**	**9,02E-05**	0,081708042	0,127496605	0,000159754	0,997270642
T cell NK_XCELL	**-0,468268615**	**4,48E-25**	0,007025217	0,926763432	**0,231601379**	**2,48E-06**	-0,010926616	0,881086277	**-0,244152889**	**8,04E-07**

Supplementary Table S4.

				**INVS**		**NPHP3**		**DVL1**		**DVL3**		**ANKS6**	
**PMID**	**Cancer type**	**Group**	**Drug**	**Log2 (Fold Change)**	**P value**	**Log2 (Fold Change)**	**P value**	**Log2 (Fold Change)**	**P value**	**Log2 (Fold Change)**	**P value**	**Log2 (Fold Change)**	**P value**
26997480	Melanoma	all	Anti-PD-1 (pembrolizumab and nivolumab)	-0.035	0.883	-0.156	0.5	-0.232	0.432	0.234	0.436	-0.003	0.99
26997480	Melanoma	MAPKi	Anti-PD-1 (pembrolizumab and nivolumab)	-0.095	0.901	0.397	0.686	-0.452	0.84	0.578	0.801	-0.029	0.969
26997480	Melanoma	non-MAPKi	Anti-PD-1 (pembrolizumab and nivolumab)	0.001	0.999	-0.563	0.472	-0.07	0.965	-0.019	0.991	0.004	0.996
28552987	Urothelial cancer	all	Anti-PD-L1 (atezolizumab)	0.096	0.763	-0.242	0.356	0.189	0.647	0.383	0.237	0.035	0.89
28552987	Urothelial cancer	smoking	Anti-PD-L1 (atezolizumab)	-0.128	0.945	-0.099	0.961	0.121	0.955	0.403	0.876	0.103	0.959
28552987	Urothelial cancer	non-smoking	Anti-PD-L1 (atezolizumab)	0.379	0.873	-0.423	0.87	0.279	0.922	0.365	0.917	-0.048	0.985
29033130	Melanoma	all	Anti-PD-1 (nivolumab)	0.077	0.81	-0.328	0.354	0.071	0.858	-0.142	0.745	0.776	0.0851
29033130	Melanoma	NIV3-PROG	Anti-PD-1 (nivolumab)	-0.254	0.82	-0.201	0.882	-0.017	0.99	-0.006	0.998	0.782	0.547

29033130	Melanoma	NIV3-NAIVE	Anti-PD-1 (nivolumab)	0.485	0.682	-0.425	0.767	0.116	0.94	-0.268	0.897	0.862	0.52
29301960	Clear cell renal cell carcinoma (ccRCC)	all	Anti-PD-1 (nivolumab)	-0.236	0.735	1.033	0.245	-0.097	0.937	0.321	0.817	0.259	0.783
29301960	Clear cell renal cell carcinoma (ccRCC)	VEGFRi	Anti-PD-1 (nivolumab)	0	1	0	1	0	1	0	1	0	1
29301960	Clear cell renal cell carcinoma (ccRCC)	non-VEGFRi	Anti-PD-1 (nivolumab)	0.023	0.981	1.812	0.142	-0.105	0.951	1.147	0.55	0.644	0.613
29443960	Urothelial cancer	all	Anti-PD-L1 (atezolizumab)	-0.044	0.423	**-0.223**	**0.00104**	0.028	0.746	0.089	0.114	-0.039	0.767

Response in expression of inversin or its interactome partners after treatment with various drugs modulators of inhibition of immune checkpoint (pembrolizumab, ipilimumab, nivolumab and atezolizumab) in ccRCC, urothelial cancer or melanoma (using TIMER 2.0; data from Miao et al., 2018).

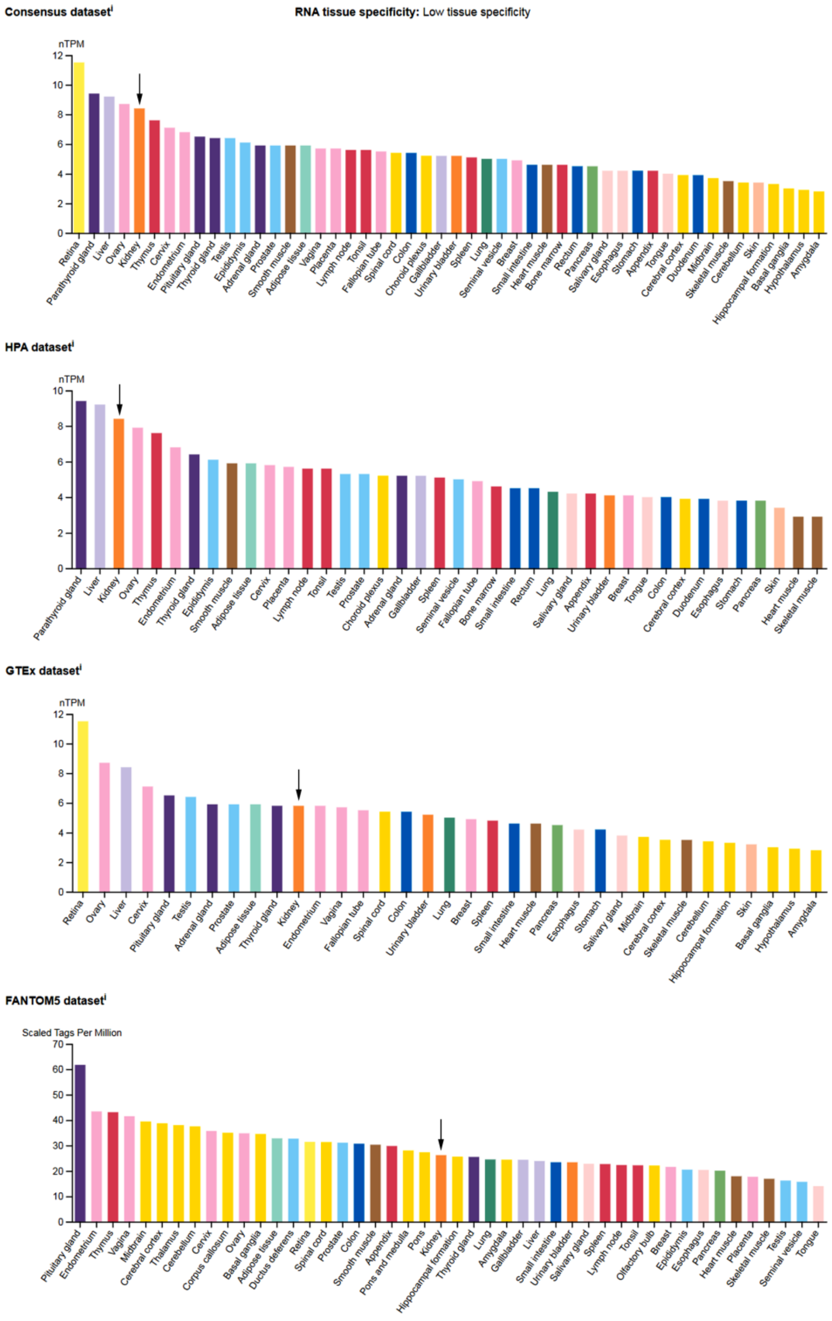

Supplementary Figure S1. Data from the Human Protein Atlas (https://www.proteinatlas.org) on the mRNA expression of inversin in different tissues, including the kidneys.

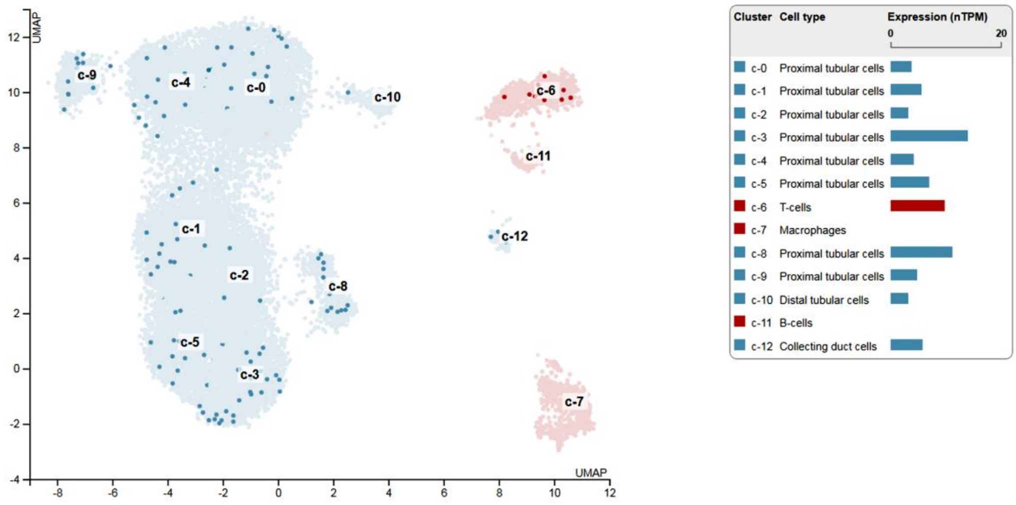

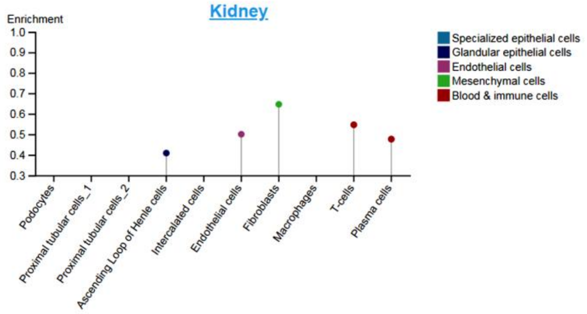

Supplementary Figure S2. Inversin mRNA expression data from the single cell transcriptome analysis of the kidney in the HPA from the GSE131685 dataset, using Human protein Atlas (https://www.proteinatlas.org/).

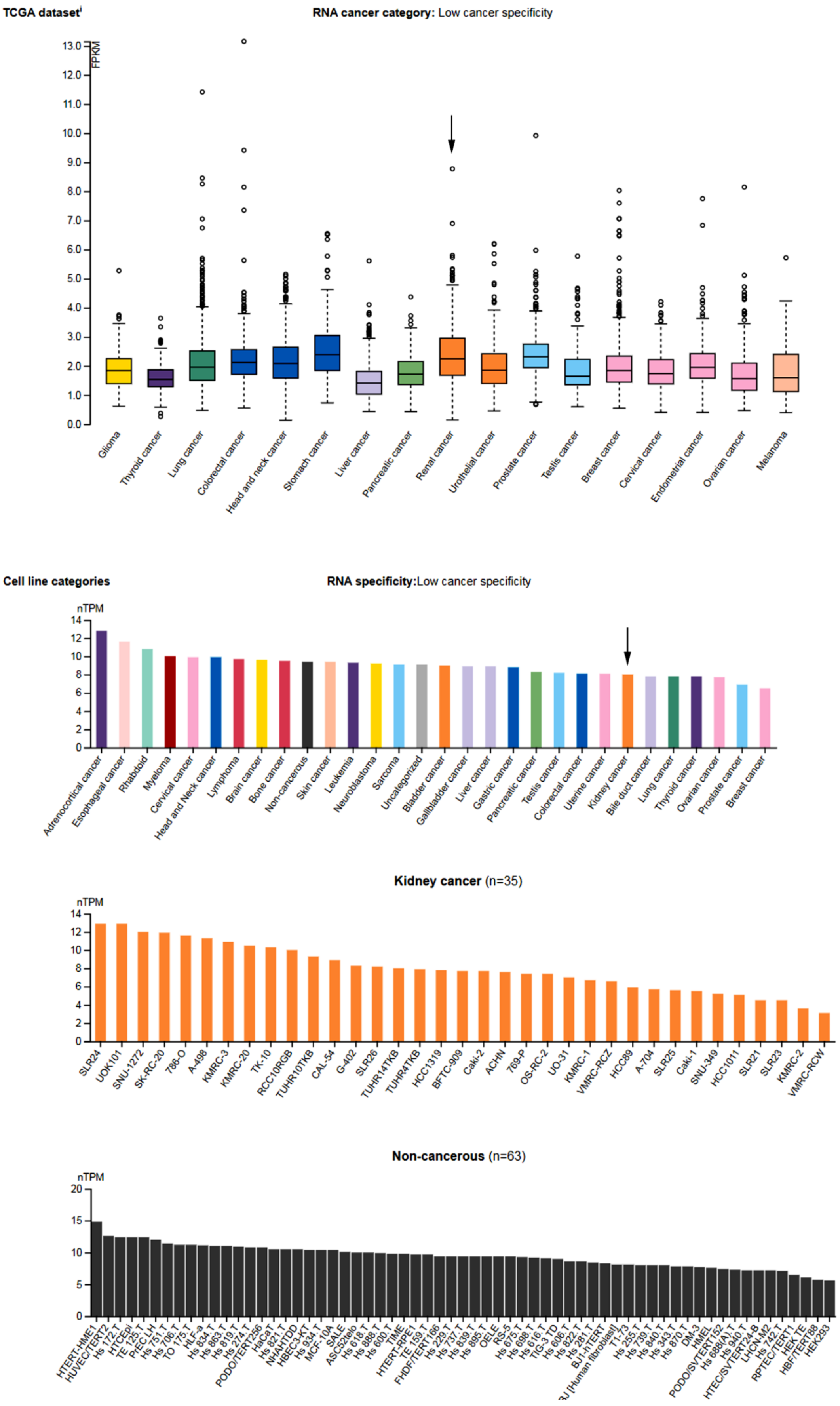

Supplementary Figure S3. Data from the Human Protein Atlas (https://www.proteinatlas.org/) on the INVS mRNA expression in different types of tumors, including ccRCC, as well as in various cell lines of normal renal cells and renal tumor lines.

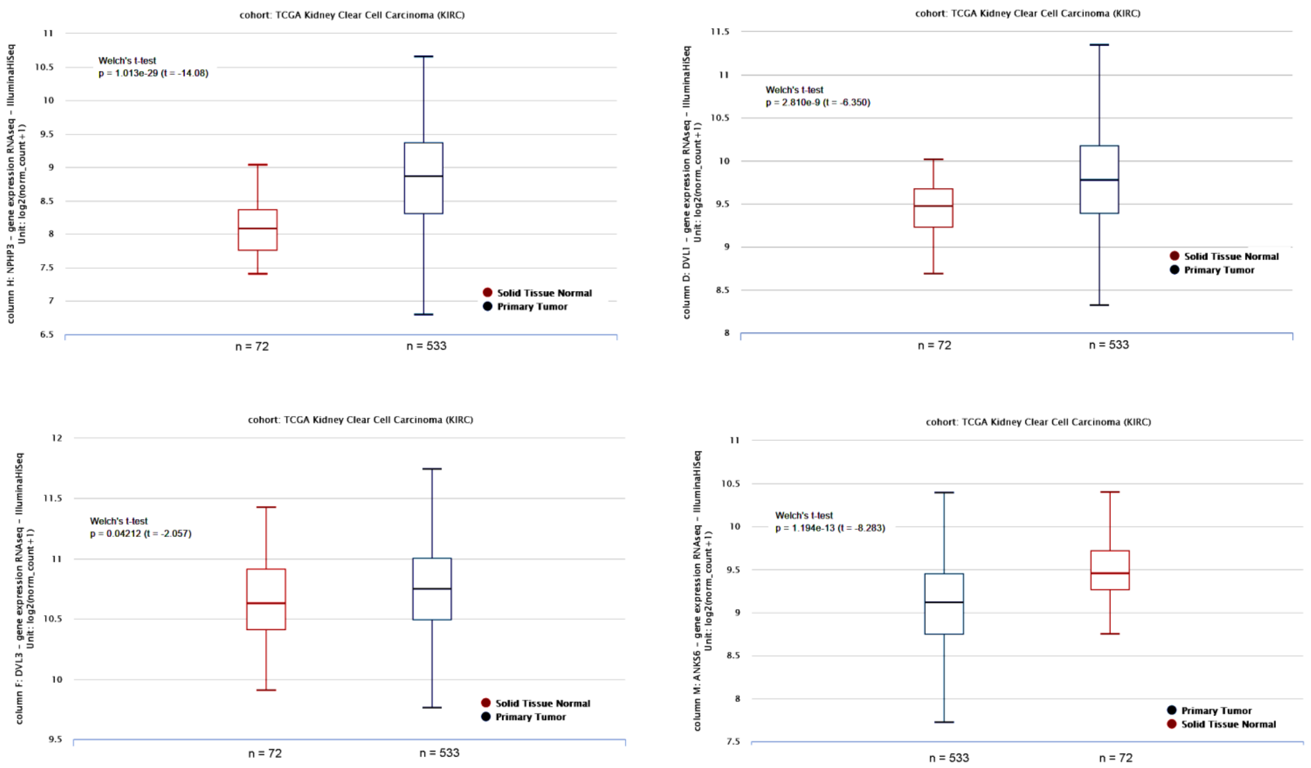

Supplementary Figure S4. The mRNA expression level of inversin interactome partners in ccRCC primary tumors and adjacent solid normal tissues in TCGA-KIRC.

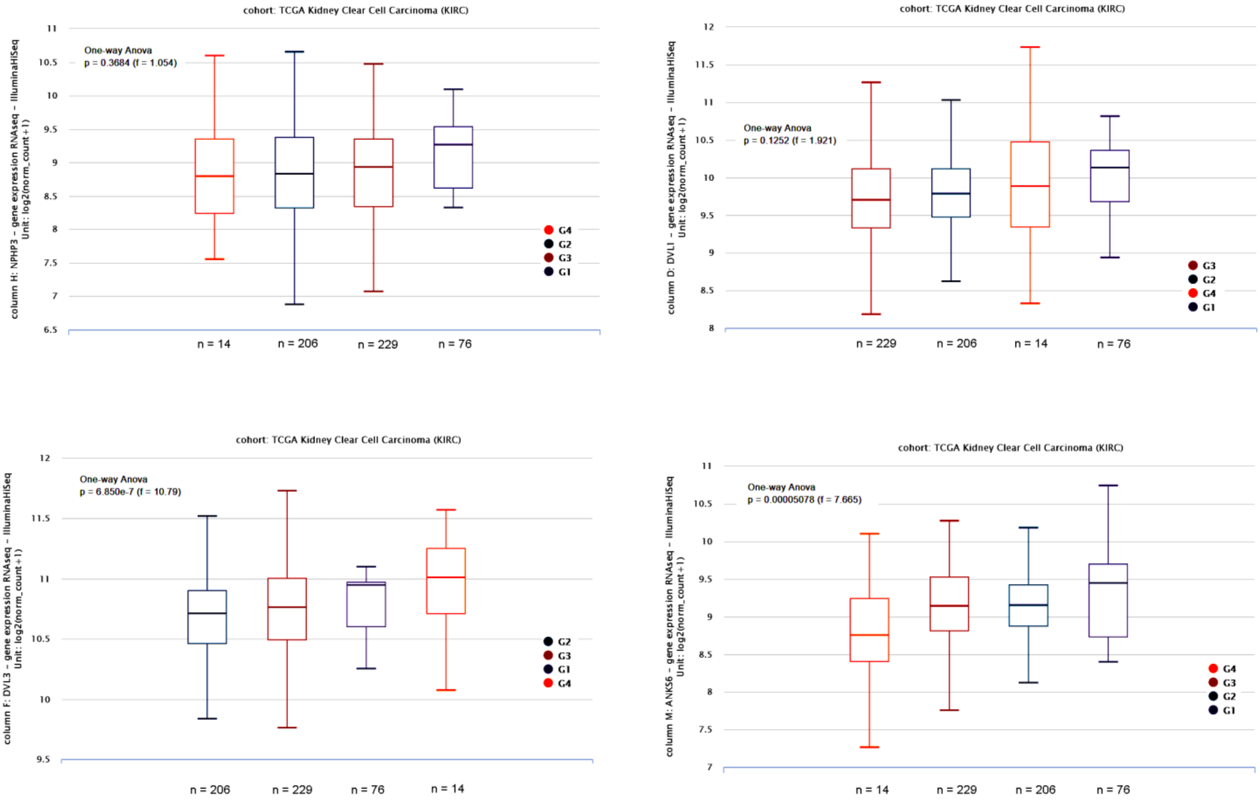

Supplementary Figure S5. Comparison of the INVS interactome partners' mRNA expression in primary tumors from TCGA-KIRC between different neoplasm histological grades.

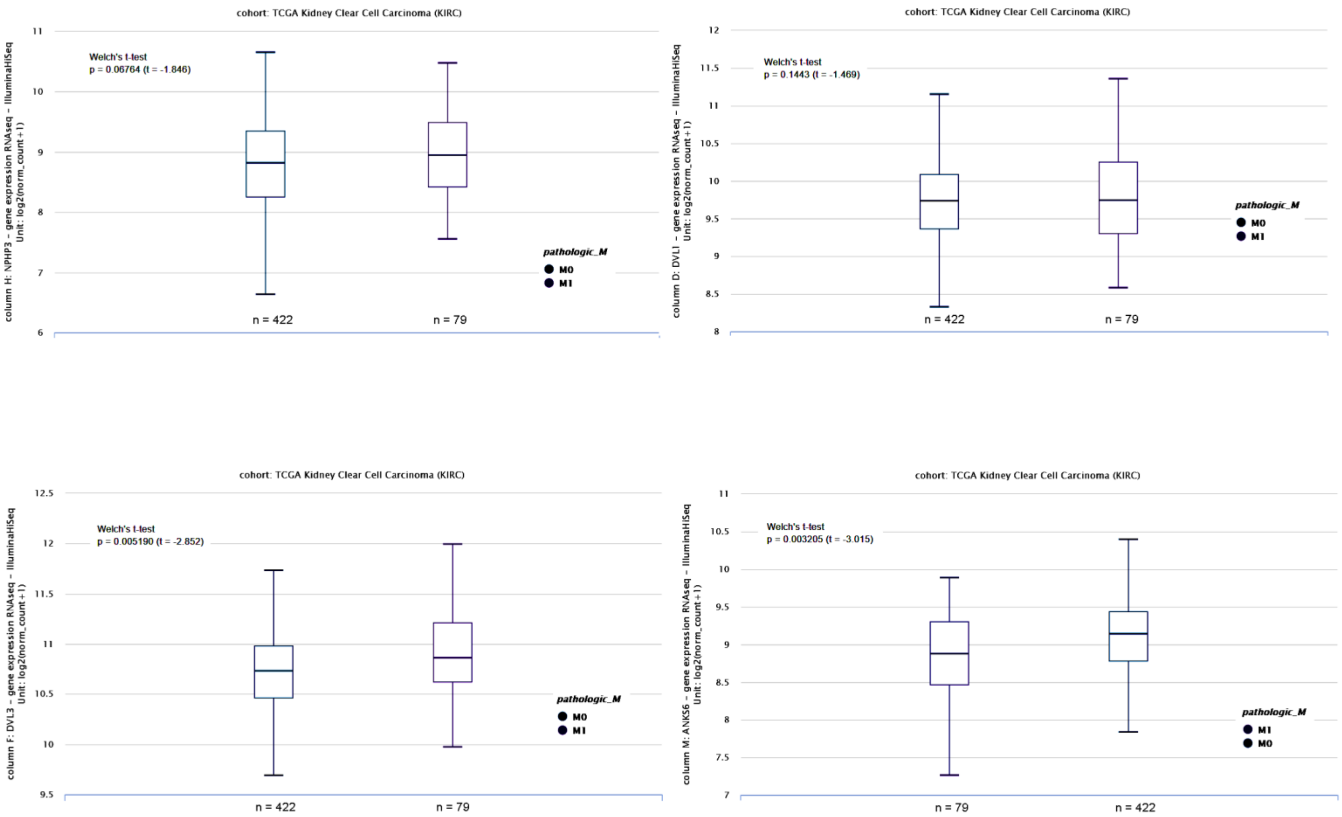

Supplementary Figure S6. Comparison of the INVS interactome partners' mRNA expression in primary tumors from TCGA-KIRC between metastatic and non-metastatic tumors.

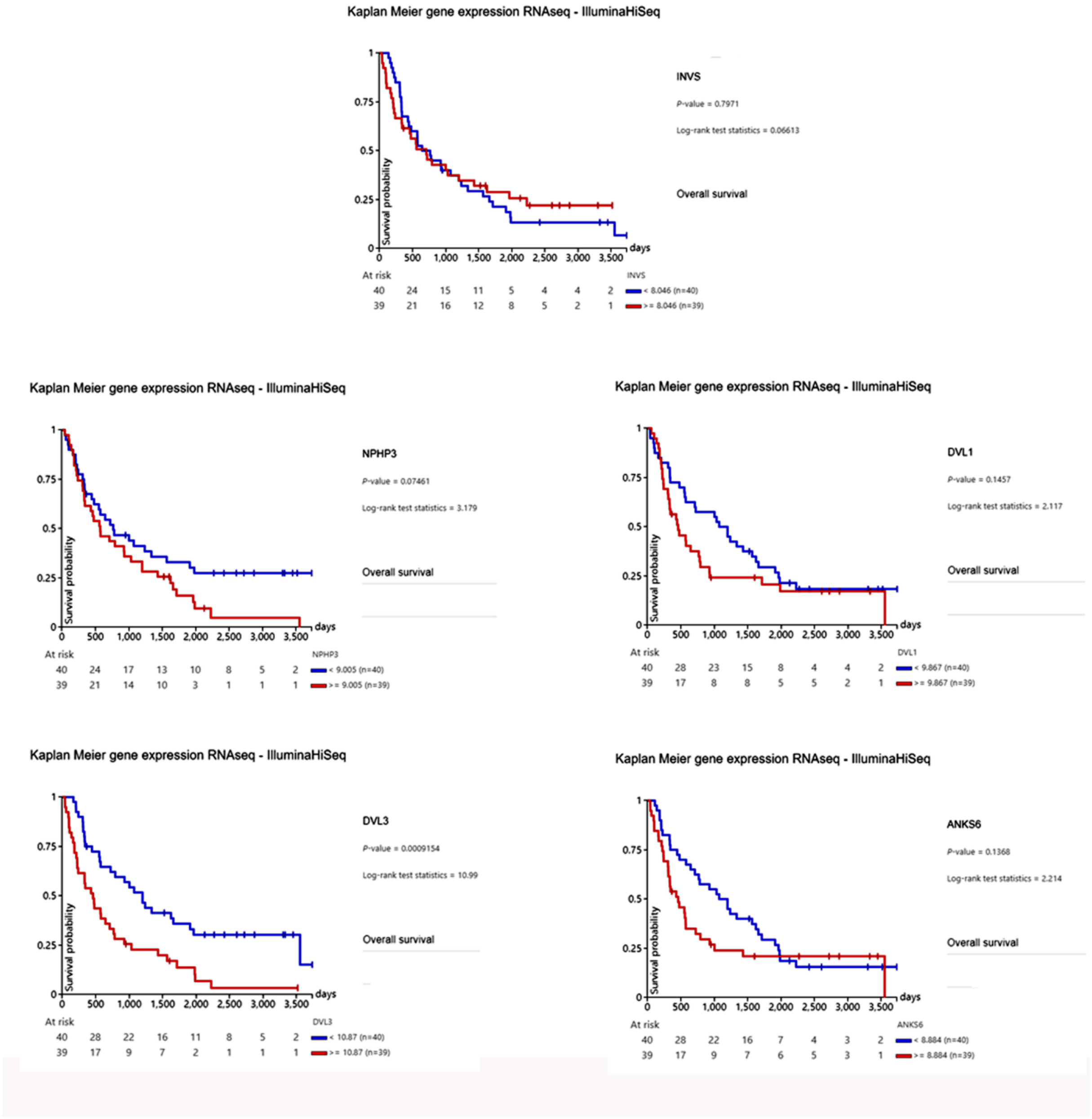

Supplementary Figure S7. The Kaplan–Meier curve analysis of the overall survival, grouped by the INVS and inversin interactome partner expression (using the median as the cutoff value) in a population of patients with metastatic tumors from TCGA-KIRC.

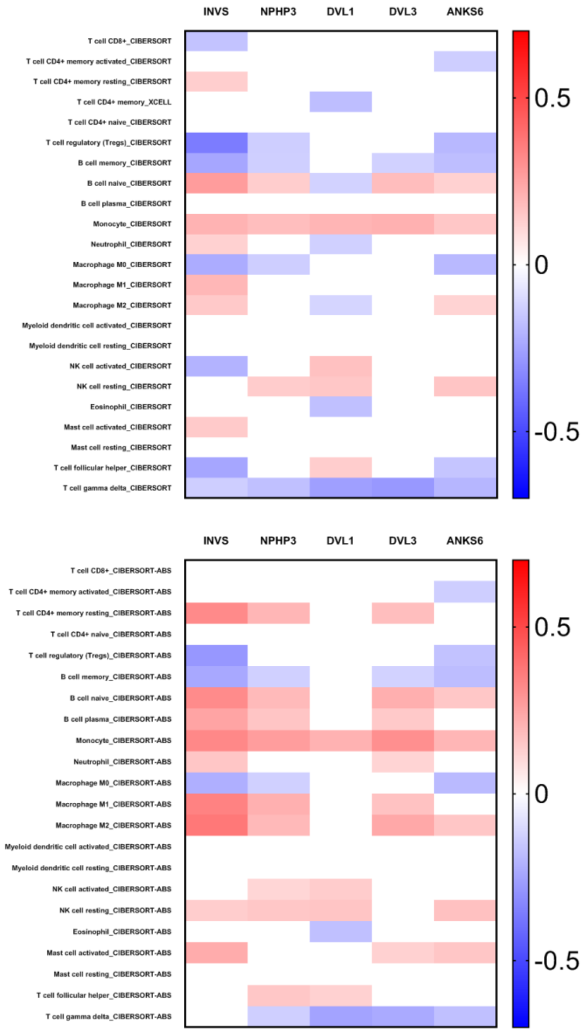

Supplementary Figure S8. Correlation analysis of expression of INVS and INVS interactome partners and abundance of tumor-infiltrating lymphocytes in TCGA-KIRC, according to the CIBERSORT and CIBERSORT-ABS algorithms. Only significant correlations are presented in color, while non-significant correlations (p>0.05) are presented in white.

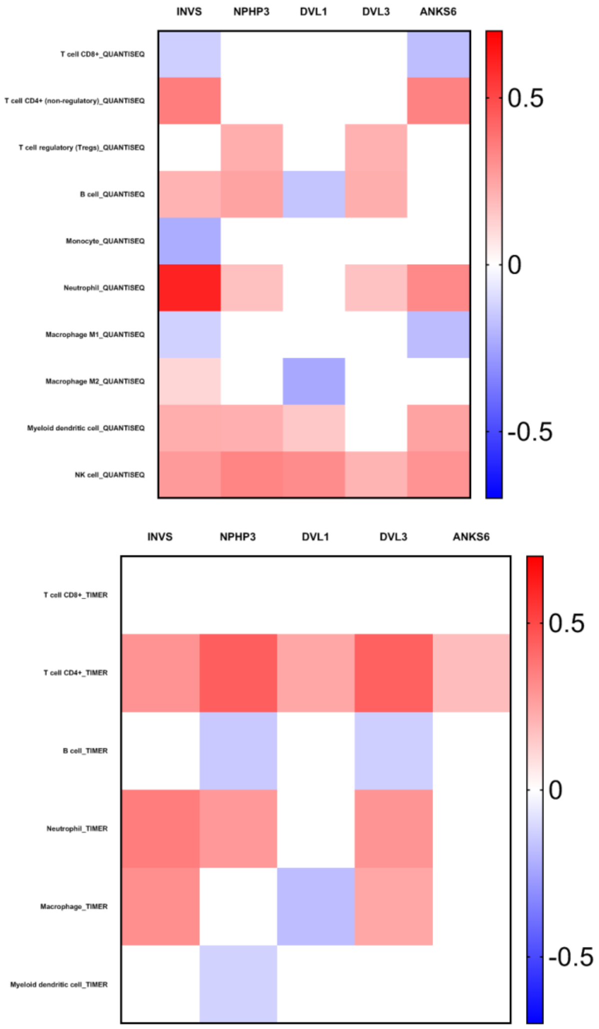

Supplementary Figure S9. Correlation analysis of expression of INVS and INVS interactome partners and abundance of tumor-infiltrating lymphocytes in TCGA-KIRC, according to the QUANTISEQ and TIMER algorithms. Only significant correlations are presented in color, while non-significant correlations (p>0.05) are presented in white.

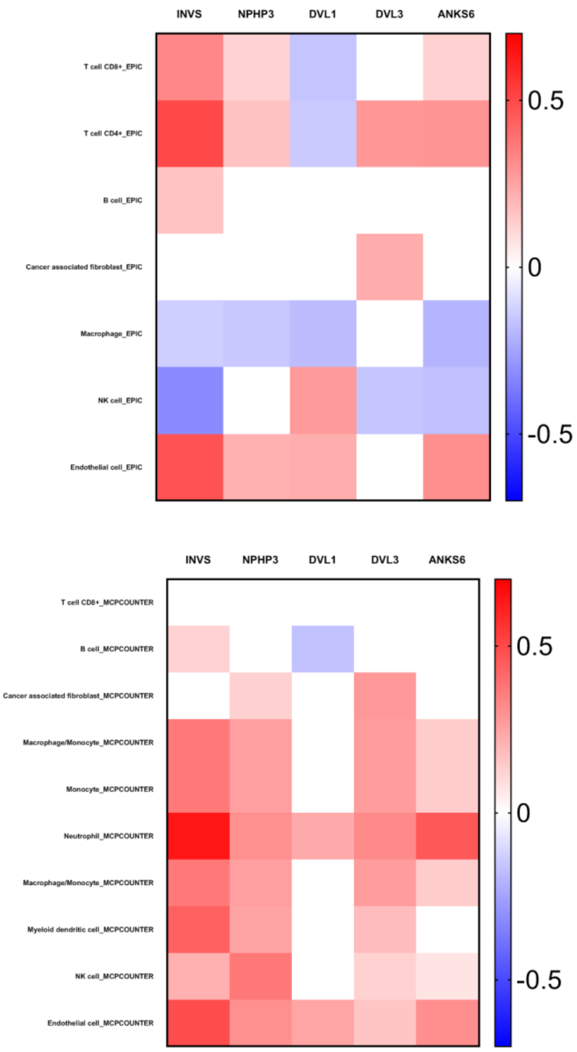

Supplementary Figure S10. Correlation analysis of expression of INVS and INVS interactome partners and abundance of tumor-infiltrating lymphocytes in TCGA-KIRC, according to the EPIC and MPCCOUNTER algorithms. Only significant correlations are presented in color, while non-significant correlations (p>0.05) are presented in white.

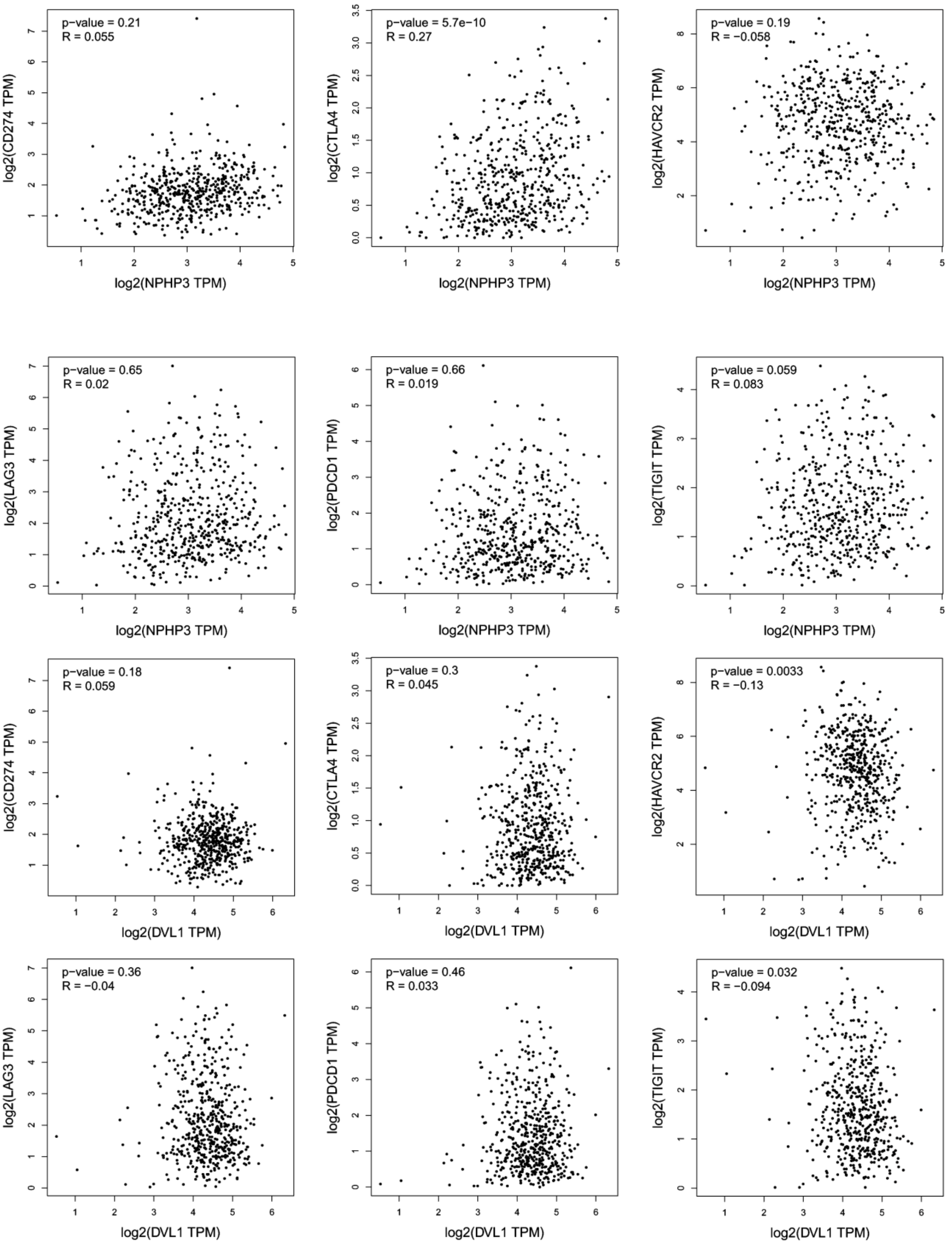

Supplementary Figure S11. The correlation of INVS interactome partner expression with immune checkpoint genes' expression in KIRC using GEPIA database.

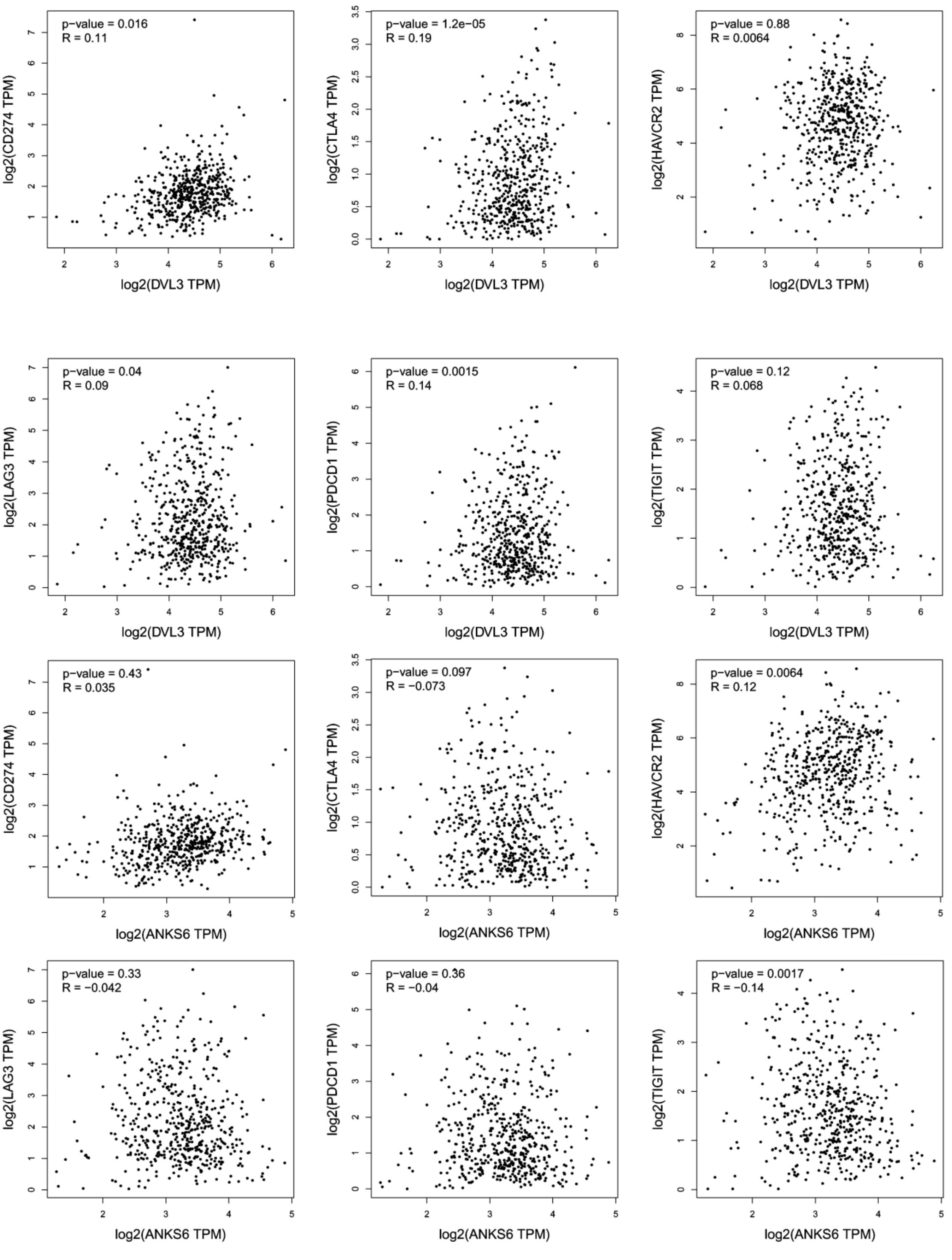

Supplementary Figure S12. The correlation of INVS interactome partner expression with immune checkpoint genes' expression in KIRC using the GEPIA database.

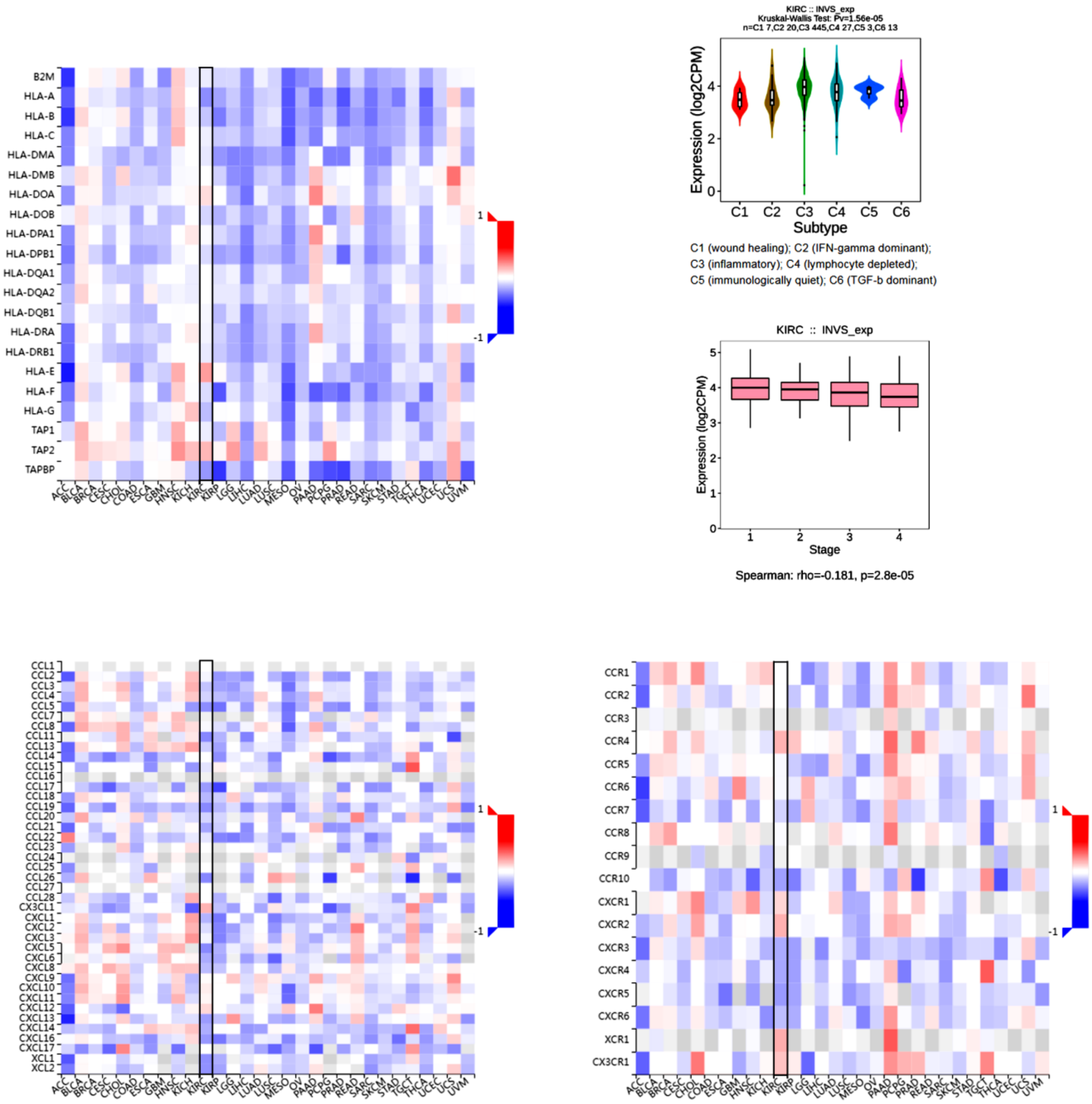

Supplementary Figure S13. Correlation analysis of INVS expression with MHC molecules, chemokines, and chemokine receptor in different cancers from TCGA (using the TISIDB database); the KIRC database is pointed out.
